# Coupling geometric morphometrics and machine learning for mandibular sex estimation in Late Pleistocene and Late Modern populations

**DOI:** 10.1038/s41598-025-31365-8

**Published:** 2025-12-19

**Authors:** Ricardo Miguel Godinho, Isabelle Crevecoeur, Susana Garcia, Rebecca Whiting, Julia Aramendi

**Affiliations:** 1 Interdisciplinary Center for Archaeology and Evolution of Human Behaviour (ICArEHB), Faculdade das Ciências Humanas E Sociais, University of Algarve, Universidade Do Algarve, Campus Gambelas, 8005-139 Faro, Portugal; 2 UMR 5199‑PACEA, CNRS, Université de Bordeaux, B8, Allée Geoffroy Saint‑Hilaire, CS 50023, 33615 Pessac Cedex, France; 3 Centre for Public Administration and Public Policies, Institute of Social and Political Sciences, MUHNAC, Universidade de Lisboa, Rua Almerindo Lessa, 1300-663 Lisbon, Portugal; 4 The British Museum, London, UK; 5 McDonald Institute for Archaeological Research, University of Cambridge, Cambridge, CB2 1TN UK

**Keywords:** Virtual anthropology, Skeletal remains, Archaeology, Morphology, Palaeodemography, Anthropology, Archaeology

## Abstract

Accurate sex estimation is crucial for studying both modern and ancient human populations, yet methods are often limited to well-preserved skeletons. Here, we combine Geometric Morphometrics (GM) and Machine Learning (ML) to assess mandibular sexual dimorphism and classify sex across a wide chronological and geographic range to bracket the potential of this approach. Sixty-seven individuals from the modern, identified Luis Lopes collection (Portugal) and 18 Late Pleistocene individuals from Jebel Sahaba (Sudan) were surface scanned. Anatomical landmark coordinates were extracted and analyzed with GM, and ML models were trained on a subset of the modern sample to predict sex in both the remaining modern individuals and the Late Pleistocene specimens. GM revealed significant sexual dimorphism in all samples, and ML achieved high intrapopulation classification accuracy. However, predictions were less reliable when applied across the temporally and geographically distant Jebel Sahaba population, reflecting interpopulation differences in mandibular size and shape. These results demonstrate that while GM–ML approaches are powerful tools for sex estimation within populations, caution is required when extending models to other populations.

**Supplementary Information:**

The online version contains supplementary material available at 10.1038/s41598-025-31365-8.

## Introduction

Sex is one of the most fundamental biological parameters assessed in forensic, biological and palaeoanthropological studies^1,2^. In addition to allowing the osteobiographical characterization of individuals, it enables ensuing analysis of, e.g., sex related differences in funerary behaviour^3–6^, weaning^7^, diet^4,8–11^, activity patterns^5,12–14^, mobility^8,13^, and pathology^15–17^.

Biomolecular methods (i.e., aDNA and proteomics) have been used to establish sex and provide very reliable results^18–27^. Moreover, such methods typically require very reduced quantities of bone/teeth, overcoming pervasive preservation issues in archaeological collections that lead to fragmentation and incompleteness of skeletal elements, often precluding reliable morphological based sex-estimation^19,21,24,25^. Yet, biomolecular methods are destructive, require highly specialized (often expensive) laboratory procedures and also depend on the preservation of proteins and/or DNA^20^. Thus, morphological-based sex estimation remains the most common and feasible approach to sex classification of archaeological individuals.

Previous morphological-based sex estimation studies have explored sexual dimorphism in most bones of the human skeleton. The most reliable regions for sex estimation are the *os coxae* and the skull, which typically provide correct classification rates above 90%^1,28–32^ (but see Spradley and Jantz ^33^ regarding the skull). Yet, these bones are often fragmented or incomplete in archaeological and/or forensic contexts. Moreover, the complexities of funerary behaviour often involve destructive procedures (e.g., cremation and reuse of funerary spaces) and/or post-depositional manipulation of the human remains, causing truncation of individuals and/or commingling and fragmentation of bones^34–42^. Thus, researchers have often to estimate sex based on individual bones rather than complete skeletons, including post-cranial bones (other than the *os coxae*) which typically provide lower correct sex classification rates and so are less reliable in sex assessment^32,43^.

Sex estimation based on the *os coxae* and skull is frequently based on scoring along semi-quantitative ordinal scales of specific anatomical regions^44–47^. While conventional metric methods are also used in these regions^48,49^, visual-based methods readily capture morphological information not easily quantifiable with the former metric approaches^44^. Yet, visual scoring is based on somewhat subjective observer specific assessment, and so some degree of inter-observer error emerges that may lead to conflicting estimations^1,45,50^. To better represent 3D morphology objectively and quantitatively, and to reduce inter-observer error, Geometric Morphometrics (GM) has been used more recently to investigate sex related morphological differences in the pelvis^51–53^ and skull^54–58^. These approaches have provided very good results, but the use of conventional landmarks (LMs) is limited in capturing 3D morphology. Hence, some studies have also used semi-sliding landmarks to enable dense coverage of the cranium and to provide better morphological representation^59^.

Machine learning (ML) has also been recently used in sex estimation. Several types of data have been used to create ML models, including linear measurements from cranial^60,61^ and post-cranial bones^62–67^, and cross-sectional data from long bones^68^. While ML often improves correct sex identification, it has seldom been applied together with GM methods^59,69^. Moreover, to the best of our knowledge, no studies have used ML models developed with identified collections and tested sex classification of archaeological specimens with sex already previously estimated based on multiple skeletal regions (including pelvises, crania and mandibles). This is particularly relevant to test the potential and limitations of applying ML models to estimate sex in archaeological specimens and so to enable examination of sex-based differences in past populations. This is the case of mandibles, which are often used in studies about the morphological impact of population history and diet on past populations^70–75^. Because they are often found isolated from the remaining skeleton, sex information is typically not estimated due to uncertainty of predictions. Thus, it is vital to enhance mandibular sex estimation reliability to enable further examination about past populations.

Here we use 3D GM to capture the mandibular morphology of an identified skeletal collection (Luis Lopes) and examine sex differences. We then use resulting outputs to train ML models, classify the sex of held-out specimens from the same (Luis Lopes) population and quantify the reliability of the ML predictions. Further, we use those models to classify Late Pleistocene mandibles from Jebel Sahaba which have been previously sexed morphologically (based on multiple skeletal regions) to examine the reliability of the GM based ML sex classifications. The selection of such diverse testing samples is deliberate and aims to bracket the reliability of this sex estimation approach. Specifically, the Luis Lopes intra-population testing sample is expected to provide the most reliable expectable results, whereas the Jebel Sahaba testing sample the least reliable expectable results due to its extreme (intra-specific) morphological difference (see details below).

## Results

### GM based morphological analysis

**Fig. 1:**
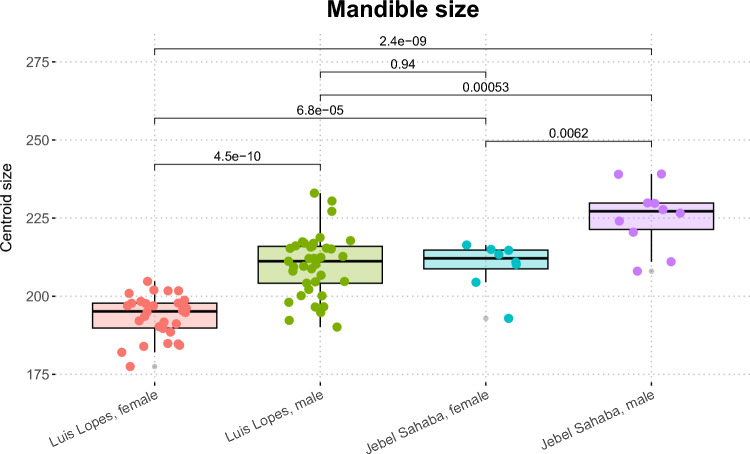
Centroid size of the specimens selected from the Luis Lopes and Jebel Sahaba collection. Results are grouped by sex and origin, with corresponding p-values of non-parametric pairwise post-hoc statistical testing. Note that all groups are significantly different from each other (except for late modern males from Portugal and Late Pleistocene females from present Sudan).

Our results show clear intra-population size differences between male and female mandibles. Within each population, male mandibles are clearly larger than those of females (Fig. 1). The archaeological specimens from Jebel Sahaba are, however, larger than those from late Modern Portugal, with no statistically significant differences between females from the former and males from the latter.

**Fig. 2:**
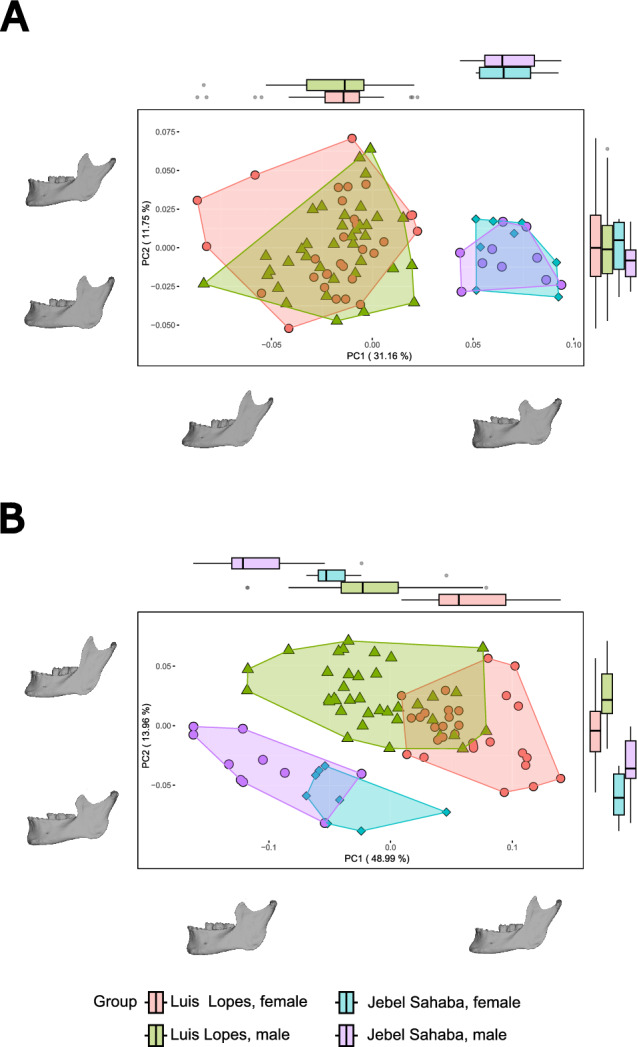
(**A**) Shape and (**B**) form PCAs. Results are colour coded and grouped by sex and origin. Note that form space shows apparent intra-population sex differences. In shape space there are no apparent intra-population sex differences, but clear inter-population differences.

The statistical analyses show morphological differences between populations and between the sexes. Specifically, the PERMANOVA including the first PCs accounting for ~ 95% of the total variance shows statistically significant differences between all groups (males and females originating from Portugal and Sudan) in form space. In shape space, all groups are significantly different, with the exception of males and females from Jebel Sahaba (Table SI 1). Such differences are apparent in the PCA plotting PC1 and PC2, in which there is a clear distinction between the sexes of both populations in PC1 (despite some overlap) in form, but not in shape space, in which PC1 separates the two populations (Fig. 2). Males (which display lower PC1 scores within each population) have more robust mandibles in form space in both samples, with, e.g., more vertical mandibular symphyses, broader and upright rami and wider sigmoid notches compared to females (which display higher PC1 scores within each population). PC1 in shape space, which separates the two populations, shows that the Jebel Sahaba mandibles are much more robust than those from the late modern Luis Lopes specimens.

As expected, the space that includes size (form) is strongly correlated with size Table SI 2). In shape space the first (and some ensuing) PC is also tightly correlated with size when including both populations in the analysis. However, when the populations are separated, the first PCs are no longer correlated with size within each of them, suggesting the relationship between some of the shape PCs and size in this space are driven by the size differences between the populations.

### Machine learning

Multiple ML models were trained with different datasets to examine which would produce the best results both in shape and form space. Specifically, the models were run using eight different sets of PCs in shape and form space, including those capturing 100% of the total variance (56 PCs for shape and 57 PCs for form), 95% of the total variance (23 PCs for shape and 18 PCs for form), 90% of the total variance (16 PCs for shape and 12 PCs for form), and PCs considered significant with a value below 0.05 (Figure SI 1).

**Fig. 3:**
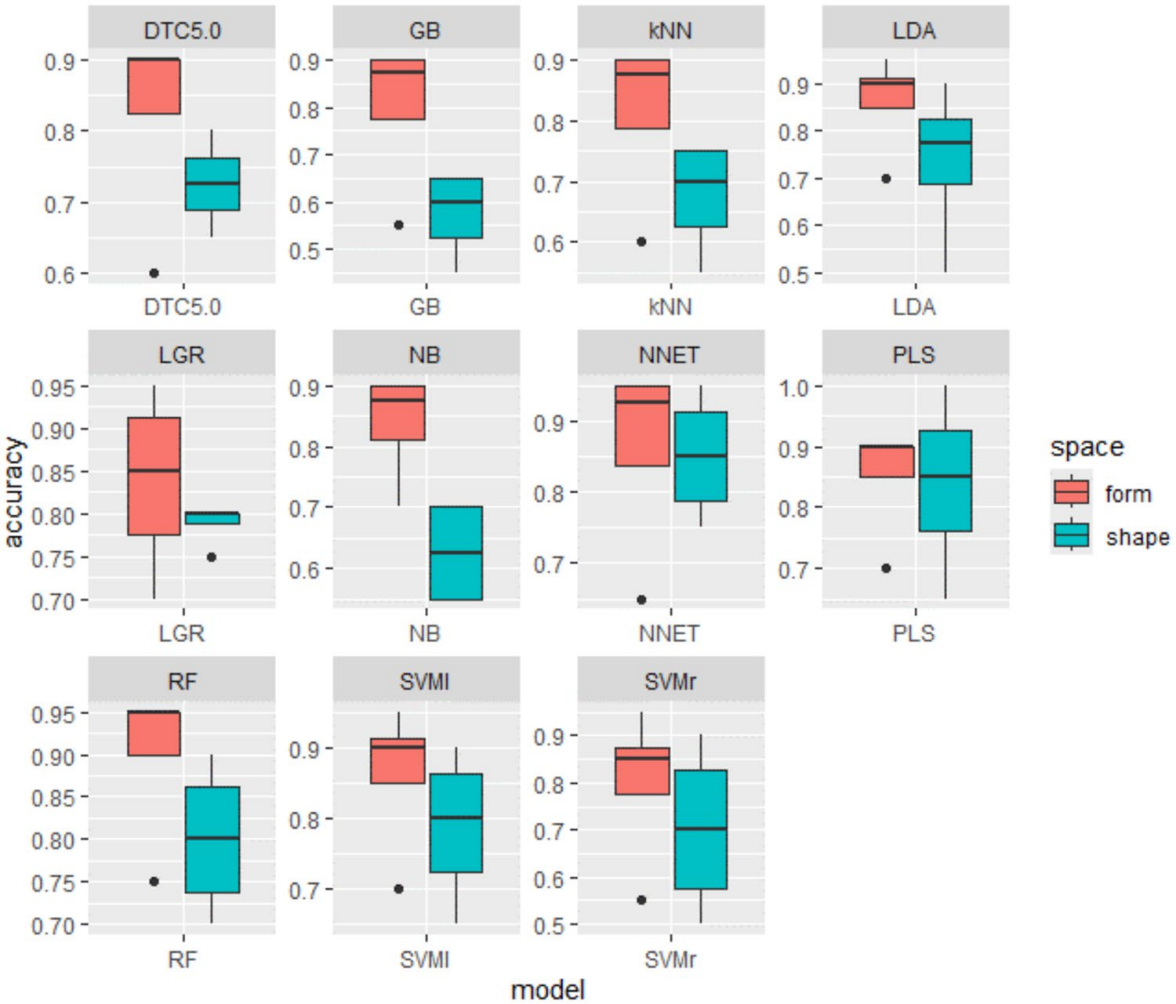
Accuracy achieved by the best-performing ML models trained with the different sets of PCs (including 100%, 95%, 90% of the total variance and the PCs established based on p values) in shape and form created for this particular study.

Model performance varies depending on the number of PCs included in the analyses. According to our results, models generally perform best when using 90% of the total variance (Table 1), while they perform the worst when using the full set of PCs obtained after PCA (Table SI 3). The latter result is likely due to the inclusion of too many residuals in the analyses. Nonetheless, perfect accuracy was achieved only when using 95% of the variance in shape (Table SI 4). With 90% of the total variance, accuracy rates range from 65 to 95% (Table 1), though the most common accuracy rate is 90%, with analyses on form variables generally performing better than those on shape. This trend is observed across all analyses (Fig. 3, Table SI 4 and Table SI 5), suggesting that size differences may be a significant factor in distinguishing male from female individuals in modern *Homo sapiens*. This is consistent with the size results above, which show significant size differences between males and females in both samples.

**Table 1:** Results provided by ML algorithms based on PCs accounting for over 90% of the total variance in shape and form.

Model	Data	Accuracy	Kappa	AccLower	AccUpper	Sensitivity	Specificity	BalAccuracy
**kNN**	shape	0.75	0.5	0.509	0.9134	0.7778	0.7273	0.7525
	form	0.85	0.7059	0.6211	0.9679	1	0.7273	0.8636
**LGR**	shape	0.8	0.596	0.5634	0.9427	0.7778	0.8182	0.798
	form	**0.9**	**0.798**	**0.683**	**0.9877**	**0.8889**	**0.9091**	**0.899**
**DTC5.0**	shape	0.65	0.3	0.4078	0.8461	0.6667	0.6364	0.6515
	form	**0.9**	**0.802**	**0.683**	**0.9877**	**1**	**0.8182**	**0.9091**
**RF**	shape	0.85	0.7	0.6211	0.9679	0.8889	0.8182	0.8535
	form	**0.95**	**0.9**	**0.7513**	**0.9987**	**1**	**0.9091**	**0.9545**
**GB**	shape	0.65	0.2857	0.4078	0.8461	0.5556	0.7273	0.6414
	form	**0.9**	**0.7980**	**0.683**	**0.9877**	**0.8889**	**0.9091**	**0.899**
**NB**	shape	0.7	0.4059	0.4572	0.8811	0.7778	0.6364	0.7071
	form	**0.9**	**0.798**	**0.683**	**0.9877**	**0.8889**	**0.9091**	**0.899**
**LDA**	shape	**0.9**	**0.798**	**0.683**	**0.9877**	**0.8889**	**0.9091**	**0.899**
	form	**0.9**	**0.798**	**0.683**	**0.9877**	**0.8889**	**0.9091**	**0.899**
**PLS**	shape	**0.9**	**0.7938**	**0.683**	**0.9877**	**0.7778**	**1**	**0.8889**
	form	**0.9**	**0.802**	**0.683**	**0.9877**	**1**	**0.8182**	**0.9091**
**SVMl**	shape	0.85	0.7	0.6211	0.9679	0.8889	0.8182	0.8535
	form	**0.9**	**0.7938**	**0.683**	**0.9877**	**0.7778**	**1**	**0.8889**
**SVMr**	shape	**0.9**	**0.802**	**0.683**	**0.9877**	**1**	**0.8182**	**0.9091**
	form	0.85	0.7	0.6211	0.9679	0.8889	0.8182	0.8535
**NNET**	shape	**0.95**	**0.898**	**0.7513**	**0.9987**	**0.8889**	**1**	**0.9444**
	form	**0.95**	**0.9**	**0.7513**	**0.9987**	**1**	**0.9091**	**0.9545**

Algorithms with accuracy above 90% are highlighted in bold.

**Fig. 4:**
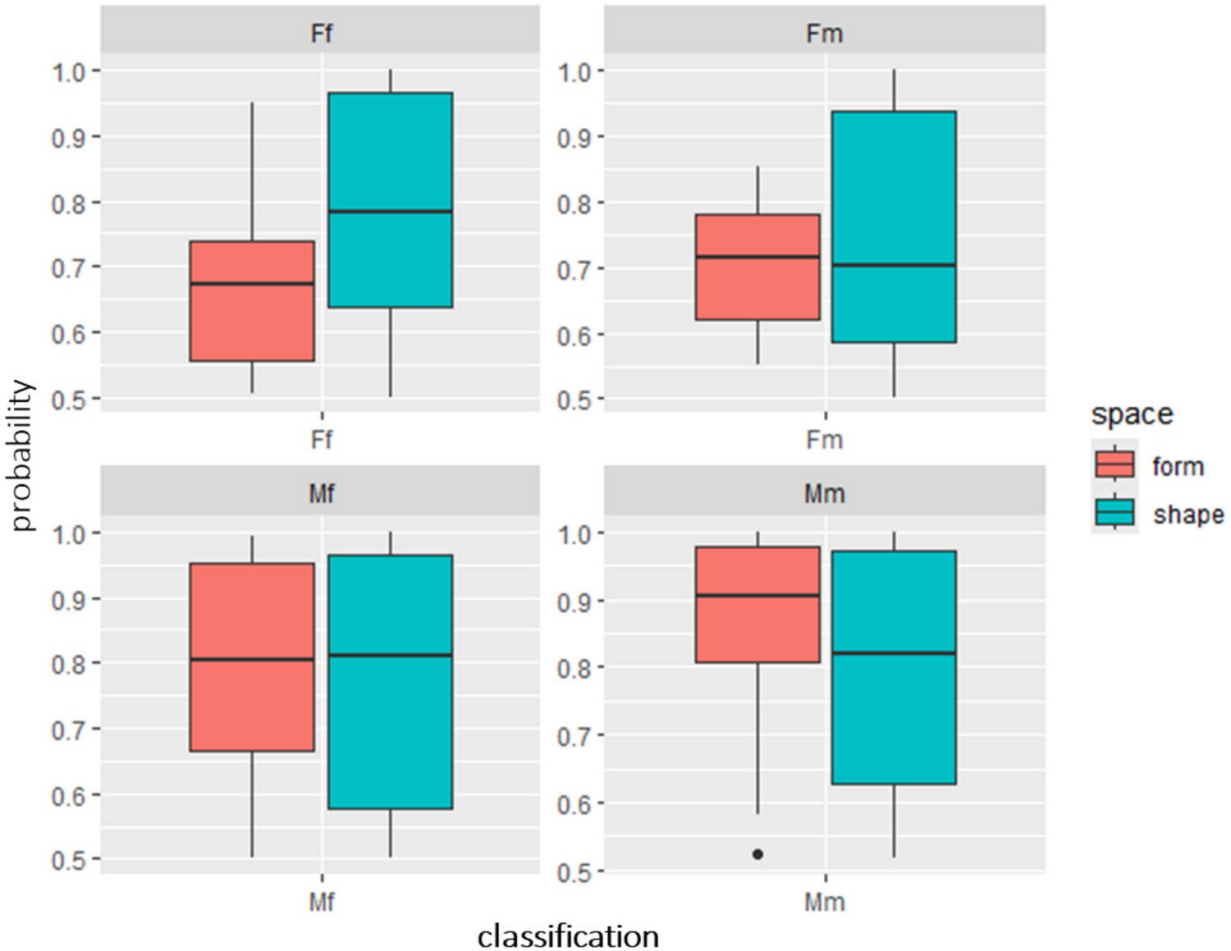
Probability percentages assigned to each archaeological sample following ML model training on the PCs capturing 90% of the total variance in both shape and form space. Classifications under *Mm* and *Ff* represent samples consistently assigned to male and female groups by both anthropological and ML methods. *Fm* denotes samples classified as female by ML models but as male by anthropological methods, while *Mf* indicates cases where ML models classify as male but anthropological methods classify as female.

The inter-population reliability of the ML models predictions was tested by contrasting the ML classifications with previous sex estimations of the Late Pleistocene Jebel Sahaba archaeological sample^76^. The results for this archaeological sample do not reach the accuracy levels achieved with the modern testing sample, assuming the skeletal morphology based anthropological classifications are correct. When using 90% of the total variance, the highest agreement between anthropological and ML-based methods is 83.33%, and the lowest is 44.44%, with a typical match rate of 61.11% (Table 2). Across the different models, there is a general tendency to overclassify the archaeological sample as male when size is included in the analyses, while classifications based on shape alone tend to be more balanced (Table 2, Table SI 6 – Table SI 8). Furthermore, correct female classifications tend to be assigned with higher confidence when using shape variables, whereas correct male classifications are more confidently made with form variables, though this trend is much more pronounced for females (Fig. 4).

**Table 2:** Classification provided by ML algorithms for the archaeological sample using the set of PCs that account for 90% of the total variance in shape and form space.

Model	Data	N male using ML methods	N female using ML methods	N same sex attribution (%)
kNN	Shape	6 (4)	12 (6)	55.56%
	Form	17 (10)	1 (1)	61.11%
LGR	Shape	13 (7)	5 (2)	50%
	Form	15 (9)	3 (2)	61.11%
DTC5.0	Shape	8 (4)	10 (4)	44.44%
	Form	17 (10)	1 (1)	61.11%
RF	Shape	6 (4)	12 (6)	55.56%
	Form	17 (10)	1 (1)	61.11%
GB	Shape	6 (4)	12 (6)	55.56%
	Form	17 (10)	1 (1)	61.11%
NB	Shape	3 (3)	15 (8)	61.11%
	Form	15 (9)	3 (2)	61.11%
LDA	Shape	9 (7)	9 (6)	72.22%
	Form	15 (9)	3 (2)	61.11%
PLS	Shape	8 (7)	10 (7)	77.78%
	Form	17 (10)	1 (1)	61.11%
SVMl	Shape	15 (9)	3 (2)	61.11%
	Form	15 (9)	3 (2)	61.11%
SVMr	Shape	8 (5)	10 (5)	55.56%
	Form	9 (8)	9 (7)	83.33%
NNET	Shape	9 (7)	9 (6)	72.22%
	Form	17 (10)	1 (1)	61.11%

The number of samples classified consistently by both anthropological methods and ML algorithms is indicated in brackets.

However, classification probabilities do not commonly exceed 90%, even for matching classifications, and high probabilities are equally common among mismatched classifications in shape space, though less frequent in form space (Table SI 9 – Table SI 16). In other analyses using different sets of PCs, match percentages between anthropological and ML methods range from 44.44% to 88.89%. The highest match rate in the study (88.89%) is obtained in form space, where the SVMr on the significant PCs appears to avoid overestimating the number of male individuals in the sample (Table SI 8).

Thus, the relationship between model accuracy on the modern sample and its performance on the archaeological sample does not appear to be straightforward. An increase in accuracy on the testing set classifications does not necessarily correspond to a direct increase in classification match for the archaeological sample. Although the accuracy of ML models trained on the modern sample is a statistically significant predictor for classifications in the archaeological sample (F < 0.05), in both shape and form, with increasing accuracy correlating with increases in the outcome variables (Figure SI 2), this association explains only a small fraction (approx. 12.5%) of the variance in the archaeological sample (Table SI 17).

## Discussion

Overall, and consistent with previous studies, our GM results show clear sexual dimorphism in mandibular morphology^77–98^, as well as inter-population morphological differences^70,71^. Consistent with previous studies using ML for sex classification^60–67^, the ML models were very efficient in the sex classification of the late modern identified Luis Lopes intra-population test sample, with average accuracies of 90% (see details above). However, the sex classification of the temporally and geographically distant mandibles of Late Pleistocene Jebel Sahaba was meaningfully less efficient, with average accuracies of 60 – 63%. Although we cannot exclude the possibility of some skeletally misclassified archaeological individuals impacting our results, these differences are most likely due to meaningful inter-population size and shape differences that resulted in frequent misclassifications (especially of females as males with form space derived data). These results are, however, predictable and consistent with previous studies highlighting inter-population morphological differences and cautioning against the use of inadequate reference samples to classify target specimens^99–104^. This is particularly relevant in this study because the individuals from Jebel Sahaba belong to a highly robust population characterized by plesiomorphic traits, extreme dental dimensions and complex crown morphology, as well as robust morphological features, some being related to powerful masticatory apparatus^105–109^. This unique phenotype has been interpreted as a consequence of population isolation during the Late Pleistocene in the Nile valley^110,111^, which may influence the classification efficiency.

The following sections discuss in more detail the GM and ML results, along with the limitations of this study and future research prospects.

### Geometric morphometrics

Our GM results show sexual dimorphism in mandibular morphology, along with inter-population differences. Males have significantly larger mandibles than females in both populations (assessed via centroid size), and shape and form sex differences were also detected. PERMANOVA also shows shape differences between males and females in the late modern identified Portuguese sample, but not in the Late Pleistocene Jebel Sahaba. In form space (which includes size) sex differences were found in both populations by the PERMANOVA. Despite these results, plotting of PC1 and PC2 in shape space shows overlapping of sexes in both populations but separation in form space. This suggests that sex differences found in lower dimensional space are mainly driven by size and that shape differences are found in higher dimensional space and in PCs which account for smaller proportions of morphological variance. This interpretation is supported by regression of PC scores against centroid size. This analysis shows that these morphological variables are (expectably) significantly related in form space in lower dimensions, and significant relationships in shape space are found only in higher dimensions. Thus, most of the sexual dimorphism we found in these samples is due to isometric size differences between sexes.

### Machine learning

ML models were trained (with supervision) using 11 different algorithms, different sets of PCs derived from both shape and form space, and were first assessed with a holdout testing sample (all using the late modern identified Portuguese sample). Overall, models performed best using the PC scores accounting for 90% of the total variance and classified sex more accurately in form than in shape space. Indeed, when using 90% of the total variance, shape-based sex estimation accuracy averaged 81%, whereas form-based sex estimation averaged 90% (see details above). These intra-population accuracies are typically higher than those reported by most studies estimating sex based on mandible morphology. Most studies report accuracies ranging from ~ 60% to ~ 85%^78,79,81–85,90–92,94,97^, with only a small number reporting accuracies of ~ 90% or more^77,87^. Further, the use of these methods mitigates inter-observer subjectivity of morphoscopic scoring^45,56,112–115^ and automates sex estimation, thus providing potentially less subjective and more reliable classifications.

Consistent with the Luis Lopes collection test sample, accuracy of sex classification of the Jebel Sahaba mandibles was higher using form than shape space derived data. However, the difference in the performance of shape and form-based models using the archaeological specimens was small. Further, the accuracy of sex classification was also much lower, averaging only 60.10% in shape and 63.13% in form space derived models (see details above). Notwithstanding, some models provided accuracies as high as 83.33% in form (SVMr) and 77.78% in shape space (PLS). The generally low accuracies in the latter space result from generally balanced misclassifications in both sexes. In contrast, females are more frequently misclassified as males in form space. Mandibles in the Jebel Sahaba sample (which have been described previously as highly robust; see above) are significantly larger than in the identified Luis Lopes collection, with females from the former presenting comparable size to the males from the latter. Thus, these misclassifications in form space are likely driven by inter-population size differences.

Consistent with previous studies, these results highlight how inter-population morphological differences impact sex estimation and may lead to potentially biased results when inadequate reference samples or methods are chosen to classify biologically distant target samples^44,99–104,116,117^. Such differences may arise due to differences in, e.g., population history^118–121^, climate^119,120^, the mechanical demands of the masticatory system^70,71,74,75,122^ and nutrition^123^. These may lead to overall interpopulation differences in size and/or shape that, in turn, impact the patterns of sexual dimorphism. Indeed, the expression of sexual dimorphism varies across populations, with contrasting patterns of robusticity in both males and females potentially leading to incorrect sex estimations when inadequate reference samples are used or estimation methods are not adjusted accordingly^100,117^. This has been hypothesized to drive the over-representation of one sex over the other in the examination of, e.g., sex ratios in past populations^124,125^.

### Limitations

Overall, this study shows that the clear sexual dimorphism in mandibular morphology can be used to estimate sex reliably within populations. However, sex classification of specimens from other populations is more challenging with meaningfully lower accuracies. This may result from several limitations of this study that will be tackled in future studies.

One of the limitations is that this study only includes one reference identified population from late modern Portugal. To classify individuals from other geographies and/or chronologies it would be very relevant to include other reference populations and so account for inter-population morphological differences. This would be particularly relevant in target samples as morphologically distinct as the Late Pleistocene Jebel Sahaba, which has been previously described as likely isolated from other populations, being particularly robust and with very large teeth (see above). Moreover, it would also be potentially relevant to increase sample size of the reference population(s) to provide a more comprehensive depiction of intra-population and sex specific morphological variance.

Conversely, this study only uses two testing samples: (i) the intra-population holdout Luis Lopes and the (ii) inter-population Late Pleistocene Jebel Sahaba samples. This choice was deliberate to enable bracketing the reliability of this sex estimation approach by using (held out) specimens from the same population used to train the ML models and an extremely morphologically distinct archaeological testing sample. This results in lower reliability results in the latter sample. Notwithstanding, we predict that ensuing studies using archaeological testing samples biologically closer to the reference sample (e.g., medieval or modern age samples originating from Portugal) will result in better results than those obtained for Jebel Sahaba.

Despite the very encouraging intra-population results using the LM dataset adopted in this study, no semi LMs were used. The use of the latter would provide a more detailed morphological representation of the specimens and of specific anatomical regions known to show significant sexual dimorphism (e.g., chin, gonial angle, posterior ramus). This may enable better sex classification accuracies. However, the application of such LMs to archaeological specimens will be challenging frequently. This is because archaeological specimens are often fragmented, precluding the use of dense landmarking protocols.

The sex of the specimens from the Late Pleistocene Jebel Sahaba sample was previously estimated using standard multi-factorial anthropological methods, including pelvis and skull-based sex estimation^76^, and this classification was used as reference to assess the reliability of the ML predictions. Although pelvis and skull-based sex estimation typically provides very reliable results, we cannot exclude the possibility that some of the more incomplete individuals may have been misclassified also due to the inevitable absence of reference populations similar to the Late Pleistocene Jebel Sahaba. Thus, the use of modern reference data may drive misclassification in targeting biologically and morphologically distinct populations^125^. If this was the case, this would impact our results. To overcome this limitation, future studies will include archaeological samples for which sex is estimated independently using biomolecular methods (i.e., aDNA or palaeoproteomics). This powerful approach will allow sex determination of the archaeological individuals and ensuing creation of archaeological reference samples. These will account for geographical and temporal differences and enable the development of archaeological population specific morphological sexing methods.

## Materials and methods

This study is based on 85 adult mandibles (Table 3). Sixty-seven originate from the Lisbon Luís Lopes identified skeletal collection^126^ and the remaining 18 from the Jebel Sahaba Late Pleistocene sample^76^. The selected specimens were surface scanned to enable GM based morphological analysis. To that end, landmark coordinates were extracted from the generated meshes for ensuing use in standard GM analysis. GM outputs were then used in supervised ML to create models that enable the mandibular-based prediction of sex in the archaeological Jebel Sahaba sample.

**Table 3:** Sample composition of the individuals selected from the collections used in this study.

	Female	Male	Total
Luis Lopes (Portugal)	30	37	67
Jebel Sahaba (Sudan)	8	10	18
Total	38	47	85

### Specimen selection

Only individuals no younger than ~ 18 years of estimated age were used in this study. This is because mandibular morphology diverges between males and females during puberty, and so younger individuals do not present meaningful sex-related morphological differences^127^. Further, growth and development induce major morphological changes that are not of interest in this study and that would obscure the sex-related morphological differences^127^. Age assessment of the individuals (i.e., non-adult vs. adult) from the identified collection was based on the records of the individuals and validated via scoring of dental development and eruption sequences^128^. This was particularly suitable as completion of dental development plays a role in the final shape and size of the mandible. The sex identified on the individual records of the Luis Lopes collection (curated by the co-author SG) was used for the GM analysis of sexual morphological differences and in the validation of the ML predictions. The age at death (i.e., non-adult vs. adult) of the archaeological individuals from Jebel Sahaba was estimated via direct observation of dental eruption sequences^128^. The sex of these individuals was previously estimated by Crevecoeur, et al. ^76^ based on the observation of the hip bones (Bruzek^129^, Murail et al.^130^ and Bruzek et al.^31^) and the skull (Buikstra and Ubelaker^46^), with estimation reliability reported therein. These estimations by Crevecoeur, et al. ^76^ were used on the GM based examination of sex related morphological differences and on the ML predictions of sex.

Selection was also restricted to specimens that were fully, or almost fully, complete for the landmarking protocol used in this study. This option was favoured because estimation of the original location of the anatomical LMs introduces some degree of error and, so, noise to the analysis (see details below). Thus, 53/67 (79.1%) mandibles presented no missing LMs, and only the remaining 14/67 (20.9%) presented one (9/67; 13.4%) or two (5/67; 7.5%) missing LMs in the Luís Lopes identified sample (Table SI 18). In the Late Pleistocene archaeological sample from current Sudan, 4/18 (22,2%) mandibles were complete, 5/18 (27,8%) had one missing LM, 1/18 (5,6%) 2, 5/18 (27,8%) 3, 2/18 (11,1%) 4 and 1/18 (5,6%) 5 missing LMs (Table SI 18). This strict selection criteria resulted in the exclusion of several individuals due to fragmentation or oral pathologies impacting morphology (e.g., extensive ante-mortem tooth loss).

### Digitization and GM based morphological analysis

**Fig. 5:**
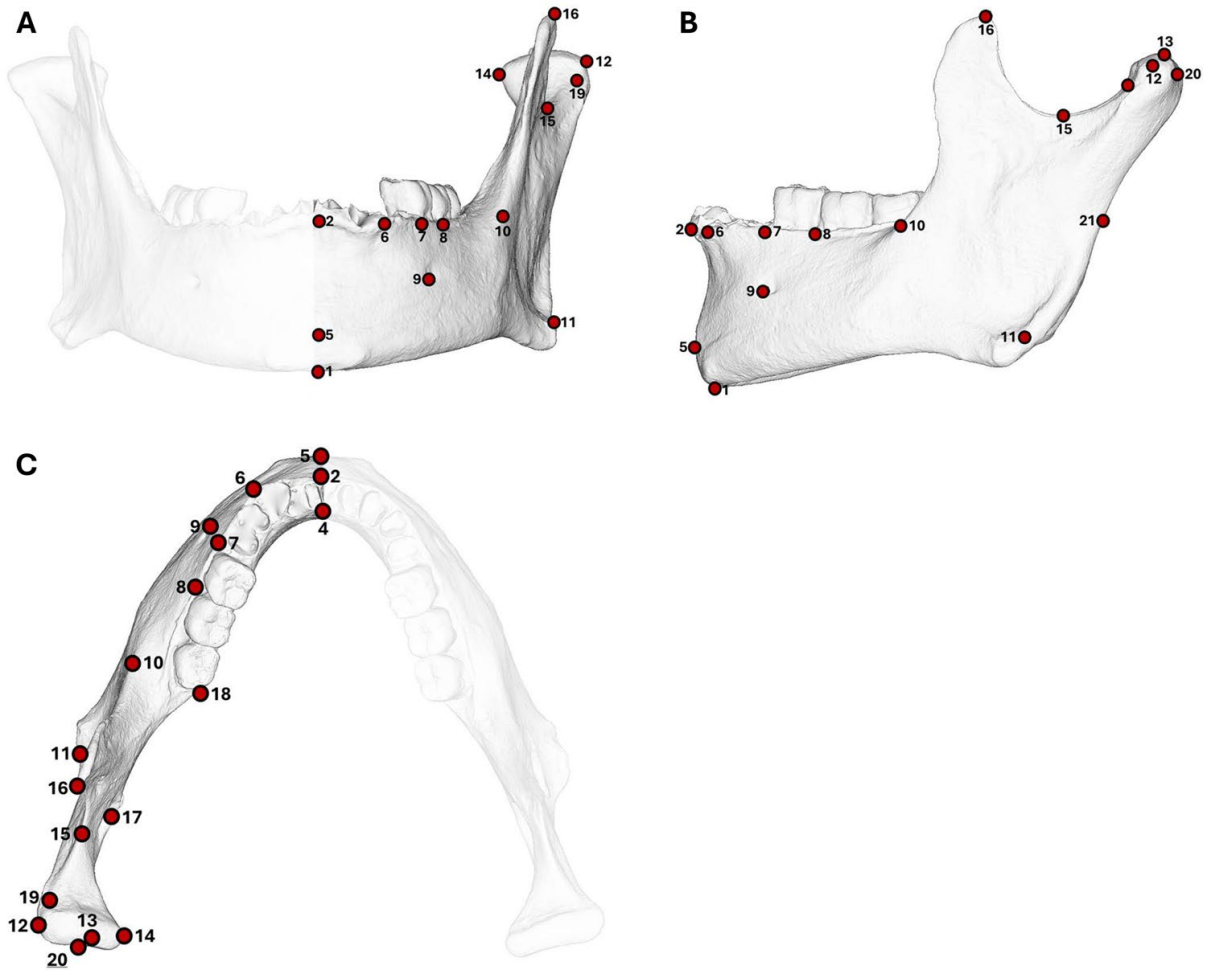
Landmarks used for extraction of coordinates. Specimen in (**A**) anterior, (**B**) lateral and (**C**) superior view.

After selection, specimens were digitised using an Einscan Pro 2X Plus structured light surface scanner. Points clouds were converted into meshes using the EXScan Pro software, which were then used to collect coordinates from a set of 21 anatomical landmarks per specimen (Fig. 5 and Table SI 19). The LM coordinates were collected in the open-source 3D Slicer software^131^.

The coordinates were then imported into R and the packages Geomorph^132^ and Morpho^133^, were used for ensuing reconstruction of incomplete specimens and GM analysis. The original location of missing anatomical landmarks was estimated using the *estimate.missing* Thin Plate Splines (TPS) based function of Geomorph. TPS based reconstruction provides reliable predictions of the original morphology but, nonetheless, introduces some errors. The magnitude of the errors relates to which and how many LMs are being reconstructed^134^. While we restricted the maximum number of missing LMs to 5, most of the selected specimens displayed the complete set of (or lacked up to a maximum of two) LMs (Table SI 18). Previous studies have also shown that significant estimation errors may emerge when inadequate specimens are selected as reference^135–137^. Although we found no significant error differences when selecting reference specimens from different modern human populations in another study^70^, we opted for population specific missing data estimation. (i.e., incomplete specimens from the Portuguese sample were reconstructed based on complete specimens from Portugal; incomplete specimens from Jebel Sahaba were reconstructed based on complete specimens from the same population).

Two spaces were used in the GM analysis: shape and form. In shape space, scaling removed (isometric) size differences^138–140^. In form space, (isometric) size differences were first removed via scaling and were then re-introduced via inclusion of log centroid size^138,141^. Differences in size between the sexes were examined using Centroid Size (CS). Principal Component Analysis (PCA) ensued to examine shape and form differences between males and females, which were visualized together with TPS warpings along the relevant principal components (PCs). Shape and form PC scores were also regressed against size (i.e., centroid size) to examine the relationship between these variables for the whole pooled sample and in each individual population.

A Kruskal Wallis test, ensued by post-hoc tests, was used to examine hypothetical differences in size (as assessed via centroid size) between sexes and populations. PERMANOVA was performed in Past^142^ to test for shape and form statistical differences between males and females using the PC scores of the first PCs explaining ~ 95% of the total variance.

### Machine learning

Principal components scores derived from shape and form analyses were subjected to ML methods. Initially, the late modern Luis Lopes identified sample was split into two sets: training (70%) and testing (30%), to assess model reliability. Analyses were conducted in shape and form space across four rounds: using the full variance, PC scores accounting for 95% of total variance, PC scores accounting for 90%, and PCs identified as significant by permutations with the GraphGMM library^143^.

ML models require large sample sizes for effective training, so artificial methods to increase sample size are commonly used in archaeology, paleontology, and anatomy^144–146^, as these fields are often limited by access to reference collections, as is the case in the present study. However, no artificial sample-increasing methods were used in this study. Bootstrapping was avoided, as it merely duplicates known data multiple times, potentially leading to overfitting during model training^146,147^. Similarly, given that our sample is limited to a specific population, applying generative adversarial networks to generate artificial data within the morphological variance range was deemed unsuitable^146^. Therefore, we proceeded with only the collected sample, with the aim that additional sexed osteological collections from different geographical areas and chronologies will eventually be incorporated into the training set in future studies.

Among ML approaches, supervised learning methods were preferred, requiring a pre-classified dataset with built-in learning and self-control mechanisms. To mitigate potential overfitting, self-correcting techniques such as k-fold cross-validation were employed. The original sample was partitioned into 10 sets to generate “submodels” with performance across these submodels assessing overall model efficiency.

The sexed modern osteological Luis Lopes collection was used to train the models, which were then applied to classify Late Pleistocene mandibles as male or female. Eleven algorithms were used in this study, including k-Nearest Neighbour (kNN), Logistic Regression (LGR), Decision Trees (DTC5.0), Random Forest (RF), Gradient Boosting (GB), Naïve Bayes (NB), Linear Discriminant Analysis (LDA), Partial Least Squares (PLS), Linear and Radial Support Vector Machines (SVMl and SVMr), and Neural Networks (NNET). All algorithms were trained using the ‘caret’^148^ and ‘caretEnsemble’ ^149^ R libraries. Model tuning, essential for classification accuracy, was conducted using hyperparameter grids to test various parameter values, with optimal values selected for each algorithm. The ‘tuneLength’ function in these libraries enabled hyperparameter configuration by generating 20 models per algorithm, with accuracy and Kappa values guiding optimal selection.

Differences in prediction performance and classification rates among models were compared considering a set of factors, including kappa, sensitivity, specificity and balanced accuracy values. Kappa statistics account for chance in predictions, with values ranging from -1 to 1 (kappa > 0.8 signals high predictive power)^150^. Sensitivity and specificity measure opposite rates: true positive versus true negative classifications^150^. These values are balanced through averaging, resulting in a model efficiency score from 0 to 1^151^.

Classifications for the archaeological sample were compared across models and against classifications from traditional anthropological methods based on all available skeletal elements of each individual^76^. Additionally, posterior probability values (p) were calculated for each individual to determine group membership reliability, with values exceeding 0.9 typically regarded as strong identifications.

## Permission

Access to the Luís Lopes identified sample was granted by the housing institution, the Portuguese Museu Nacional de História Natural e da Ciência (MUHNAC). Access to the Jebel Sahaba sample was granted by the housing institution, the British Museum. Both access permissions ensued formal access requests to each of these institutions and assessment by the relevant boards. All regulations of each of these institutions were followed.

## Supplementary Information

Supplementary Information.

## Data Availability

The data that support this study is available from the corresponding author upon reasonable request.

## References

[1] Krishan, K. et al. A review of sex estimation techniques during examination of skeletal remains in forensic anthropology casework. *Forensic Sci. Int.* **261**, 165.e161-165.e168. 10.1016/j.forsciint.2016.02.007 (2016). 10.1016/j.forsciint.2016.02.007 26926105

[2] White, T. D., Black, M. T. & Folkens, P. A. *Human Osteology* (Academic Press, 2011).

[3] Laffranchi, Z., Beck De Lotto, M. A., Delpino, C., Lösch, S. & Milella, M. Social differentiation and well-being in the Italian iron age: exploring the relationship between sex, age, biological stress, and burial complexity among the Picenes of Novilara (8th-7th c. BC). *Archaeol. Anthropol. Sci.* **13**, 182. 10.1007/s12520-021-01449-3 (2021).

[4] Dong, Y. et al. Shifting diets and the rise of male-biased inequality on the central plains of China during Eastern Zhou. *Proc. Natl. Acad. Sci.* **114**, 932–937. 10.1073/pnas.1611742114 (2017). 28096406 10.1073/pnas.1611742114 PMC5293112

[5] Masclans, A., Duboscq, S., Achino, K. F., Morell, B. & Gibaja, J. F. Looking for sexual differences during the middle Neolithic in the northeast of the Iberian Peninsula. *J. Archaeol. Sci. Rep.* **26**, 101858. 10.1016/j.jasrep.2019.05.023 (2019).

[6] Rebay-Salisbury, K. et al. Gendered burial practices of early Bronze Age children align with peptide-based sex identification: A case study from Franzhausen I Austria. *J. Archaeol. Sci.* **139**, 105549. 10.1016/j.jas.2022.105549 (2022).

[7] Fernández-Crespo, T. et al. Multi-isotope evidence for the emergence of cultural alterity in Late Neolithic Europe. *Sci. Adv.* **6**, eaay2169. 10.1126/sciadv.aay2169 (2020). 32010785 10.1126/sciadv.aay2169 PMC6976287

[8] Rey, L., Rottier, S., Santos, F. & Goude, G. Sex and age-related social organization in the Neolithic: A promising survey from the Paris Basin. *J. Archaeol. Sci. Rep.* **38**, 103092. 10.1016/j.jasrep.2021.103092 (2021).

[9] Rey, L. et al. A multi-isotope analysis of Neolithic human groups in the Yonne valley, Northern France: insights into dietary patterns and social structure. *Archaeol. Anthropol. Sci.* **11**, 5591–5616. 10.1007/s12520-019-00885-6 (2019).

[10] Avery, L. C., Brickley, M. B., Findlay, S., Chapelain de Seréville-Niel, C. & Prowse, T. L. Child and adolescent diet in Late Roman Gaul: An investigation of incremental dietary stable isotopes in tooth dentine. *Int. J. Osteoarchaeol.* **31**, 1226–1236. 10.1002/oa.3033 (2021).

[11] Miller, M. J., Dong, Y., Pechenkina, K., Fan, W. & Halcrow, S. E. Raising girls and boys in early China: Stable isotope data reveal sex differences in weaning and childhood diets during the eastern Zhou era. *Am. J. Phys. Anthropol.* **172**, 567–585. 10.1002/ajpa.24033 (2020). 32141612 10.1002/ajpa.24033 PMC7496748

[12] Lieverse, A. R., Stock, J. T., Katzenberg, M. A. & Haverkort, C. M. The bioarchaeology of habitual activity and dietary change in the Siberian Middle Holocene. *Human Bioarchaeol. Trans. Agric.* **18**, 263–291 (2011).

[13] Ruff, C. B. et al. Gradual decline in mobility with the adoption of food production in Europe. *Proc. Natl. Acad. Sci.* **112**, 7147 (2015). 26060299 10.1073/pnas.1502932112 PMC4466732

[14] Ruff, C. Sexual dimorphism in human lower limb bone structure: relationship to subsistence strategy and sexual division of labor. *J. Human Evol.* **16**, 391–416. 10.1016/0047-2484(87)90069-8 (1987).

[15] Bonsall, L. A comparison of female and male oral health in skeletal populations from late Roman Britain: Implications for diet. *Arch. Oral Biol.* **59**, 1279–1300. 10.1016/j.archoralbio.2014.07.019 (2014). 25150533 10.1016/j.archoralbio.2014.07.019

[16] Lukacs, J. R. Sex Differences in dental caries rates with the origin of agriculture in South Asia. *Curr. Anthropol.* **37**, 147–153. 10.1086/204481 (1996).

[17] Milner, G. R. & Boldsen, J. L. A companion to paleopathology 268–284 (2011).

[18] Lugli, F. et al. Enamel peptides reveal the sex of the Late Antique ‘Lovers of Modena’. *Sci. Rep.* **9**, 13130. 10.1038/s41598-019-49562-7 (2019). 31511583 10.1038/s41598-019-49562-7 PMC6739468

[19] Granja, R. et al. Unbalanced sex-ratio in the Neolithic individuals from the Escoural Cave (Montemor-o-Novo, Portugal) revealed by peptide analysis. *Sci. Rep.* **13**, 19902. 10.1038/s41598-023-47037-4 (2023). 37964077 10.1038/s41598-023-47037-4 PMC10646114

[20] Buonasera, T. et al. A comparison of proteomic, genomic, and osteological methods of archaeological sex estimation. *Sci. Rep.* **10**, 11897. 10.1038/s41598-020-68550-w (2020). 32681049 10.1038/s41598-020-68550-w PMC7368048

[21] Cintas-Peña, M. et al. Amelogenin peptide analyses reveal female leadership in Copper Age Iberia (c. 2900–2650 BC). *Sci. Rep.* **13**, 9594. 10.1038/s41598-023-36368-x (2023). 37414858 10.1038/s41598-023-36368-x PMC10326254

[22] Froment, C. et al. Analysis of 5000 year-old human teeth using optimized large-scale and targeted proteomics approaches for detection of sex-specific peptides. *J. Proteom.* **211**, 103548. 10.1016/j.jprot.2019.103548 (2020). 10.1016/j.jprot.2019.103548 31626997

[23] Gasparini, A. et al. Biological sex VS. Archaeological Gender: Enamel peptide analysis of the horsemen of the Early middle age necropolises of Campochiaro (Molise, Italy). *J. Archaeol. Sci. Rep.* **41**, 103337. 10.1016/j.jasrep.2021.103337 (2022).

[24] Parker, G. J. et al. Sex estimation using sexually dimorphic amelogenin protein fragments in human enamel. *J. Archaeol. Sci.*10.1016/j.jas.2018.08.011 (2018).

[25] Skoglund, P., Storå, J., Götherström, A. & Jakobsson, M. Accurate sex identification of ancient human remains using DNA shotgun sequencing. *J. Archaeol. Sci.* **40**, 4477–4482. 10.1016/j.jas.2013.07.004 (2013).

[26] Loreille, O. et al. Biological sexing of a 4000-year-old egyptian mummy head to assess the potential of nuclear DNA recovery from the most damaged and limited forensic specimens. *Genes* **9**, 135 (2018). 29494531 10.3390/genes9030135 PMC5867856

[27] Díaz-Zorita Bonilla, M. et al. Female sex bias in Iberian megalithic societies through bioarchaeology, aDNA and proteomics. *Sci. Rep.* **14**, 21818. 10.1038/s41598-024-72148-x (2024). 39313501 10.1038/s41598-024-72148-x PMC11420231

[28] Christensen, A. M., Passalacqua, N. V. & Bartelink, E. J. *Forensic Anthropology: Current Methods and Practice* (Academic Press, 2019).

[29] Luo, Y. C. Sex determination from the pubis by discriminant function analysis. *Forensic Sci. Int.* **74**, 89–98. 10.1016/0379-0738(95)01739-6 (1995). 7665136 10.1016/0379-0738(95)01739-6

[30] Meindl, R. S., Lovejoy, C. O., Mensforth, R. P. & Carlos, L. D. Accuracy and direction of error in the sexing of the skeleton: Implications for paleodemography. *Am. J. Phys. Anthropol.* **68**, 79–85. 10.1002/ajpa.1330680108 (1985). 4061604 10.1002/ajpa.1330680108

[31] Brůžek, J., Santos, F., Dutailly, B., Murail, P. & Cunha, E. Validation and reliability of the sex estimation of the human os coxae using freely available DSP2 software for bioarchaeology and forensic anthropology. *Am. J. Phys. Anthropol.* **164**, 440–449. 10.1002/ajpa.23282 (2017). 28714560 10.1002/ajpa.23282

[32] Bruzek, J. & Murail, P. *Forensic Anthropology and Medicine: Complementary Sciences From Recovery to Cause of Death* (Humana Press, 2006).

[33] Spradley, M. K. & Jantz, R. L. Sex estimation in forensic anthropology: Skull versus postcranial elements. *J. Forensic Sci.* **56**, 289–296. 10.1111/j.1556-4029.2010.01635.x (2011). 21210801 10.1111/j.1556-4029.2010.01635.x

[34] Godinho, R. M., Gonçalves, D. & Valera, A. C. The preburning condition of chalcolithic cremated human remains from the Perdigões enclosures (Portugal). *Int. J. Osteoarchaeol.* **29**, 706–717. 10.1002/oa.2768 (2019).

[35] Valera, A. C., Evangelista, L. & Godinho, R. M. *Megalithic Societies: Old Questions, New Narratives* (eds G. Higginbottom et al.) (Archaeopress, In press).

[36] Valera, A. C., Silva, A. M., Leandro, I., Godinho, R. M. & Evangelista, L. S. Os Perdigões Neolíticos. In *Génese e desenvolvimento (de meados do 4º aos inícios do 3º milénio AC)* (ed. Valera, A. C.) (Núcleo de Investigação Arqueológica (NIA), 2018).

[37] Evangelista, L. S. & Godinho, R. M. O Sepulcro 4 dos Perdigões. In *Um Tholos da Segunda Metade do 3º Milénio A.C.* (ed. Valera, A. C.) (Núcleo de Investigação Arqueológica (NIA), 2020).

[38] Sianto, L. et al. Evidence of contact between New and Old World: paleoparasitological and food remains study in the Tagus river population of Sarilhos Grandes (Montijo, Portugal). *Archaeol. Anthropol. Sci.* **10**, 75–81. 10.1007/s12520-016-0337-9 (2018).

[39] Filipe, V., Godinho, R. M., Granja, R., Ribeiro, A. & Valera, A. Bronze age funerary spaces in Outeiro Alto 2 (Brinches, Serpa, Portugal): the hypogea cemetery. *Zephyrus* **71**, 107–129 (2013).

[40] Valera, A. *et al.**4º Colóquio de Arqueologia do Alqueva - O Plano de Rega (2002–2010).* (eds António Carlos Silva, Frederico Tátá Regala, & Miguel Martinho) 55–73 (EDIA).

[41] Godinho, R. Deposições funerárias em fossa nos Perdigões: dados antropológicos do sector I. *Apontamentos de Arqueologia e Património* **3**, 29–34 (2008).

[42] Miguel, L. & Godinho, R. M. Notícia do sítio arqueológico do Monte das Covas 3 (Beja). *Apontamentos de Arqueologia e Património* **4**, 23–24 (2009).

[43] Introna, F., Di Vella, G. & Campobasso, C. P. Sex determination by discriminant analysis of patella measurements. *Forensic Sci. Int.* **95**, 39–45. 10.1016/S0379-0738(98)00080-2 (1998). 9718670 10.1016/s0379-0738(98)00080-2

[44] Walker, P. L. Sexing skulls using discriminant function analysis of visually assessed traits. *Am. J. Phys. Anthropol.* **136**, 39–50. 10.1002/ajpa.20776 (2008). 18324631 10.1002/ajpa.20776

[45] Walrath, D. E., Turner, P. & Bruzek, J. Reliability test of the visual assessment of cranial traits for sex determination. *Am. J. Phys. Anthropol.* **125**, 132–137. 10.1002/ajpa.10373 (2004). 15365979 10.1002/ajpa.10373

[46] Buikstra, J. & Ubelaker, D. *Standards for Data Collection from Human Skeletal Remains: Proceedings of a Seminar at the Field Museum of Natural History*. (Arkansas Archeological Survey, 1994).

[47] Ferembach, D., Schwidetzky, I. & Stloukal, M. Recommendations for age and sex diagnoses of skeletons. *J. Human Evol.* **9**, 517–549. 10.1016/0047-2484(80)90061-5 (1980).

[48] Santos, F., Guyomarc’h, P. & Bruzek, J. Statistical sex determination from craniometrics: Comparison of linear discriminant analysis, logistic regression, and support vector machines. *Forensic Sci. Int.* **245**, 204.e201-204.e208. 10.1016/j.forsciint.2014.10.010 (2014). 10.1016/j.forsciint.2014.10.010 25459272

[49] Techataweewan, N. et al. Metric sexual dimorphism of the skull in Thailand. *Forensic Sci. Int. Rep.* **4**, 100236. 10.1016/j.fsir.2021.100236 (2021).

[50] Colman, K. L. et al. The accuracy of 3D virtual bone models of the pelvis for morphological sex estimation. *Int. J. Legal Med.* **133**, 1853–1860. 10.1007/s00414-019-02002-7 (2019). 30680527 10.1007/s00414-019-02002-7 PMC6811666

[51] Steyn, M., Pretorius, E. & Hutten, L. Geometric morphometric analysis of the greater sciatic notch in South Africans. *HOMO – J. Comparat. Human Biol.* **54**, 197–206. 10.1078/0018-442X-00076 (2004). 10.1078/0018-442x-00076 15216665

[52] Bytheway, J. A. & Ross, A. H. A geometric morphometric approach to sex determination of the human adult Os Coxa. *J. Forensic Sci.* **55**, 859–864. 10.1111/j.1556-4029.2010.01374.x (2010). 20384930 10.1111/j.1556-4029.2010.01374.x

[53] Bilfeld, M. F. et al. Human Coxal bone sexual dimorphism and multislice computed tomography: Geometric morphometric analysis of 65 adults. *J. Forensic Sci.* **57**, 578–588. 10.1111/j.1556-4029.2011.02009.x (2012). 22211944 10.1111/j.1556-4029.2011.02009.x

[54] Kimmerle, E. H., Ross, A. & Slice, D. Sexual dimorphism in America: Geometric morphometric analysis of the craniofacial region. *J. Forensic Sci.* **53**, 54–57. 10.1111/j.1556-4029.2007.00627.x (2008). 18279240 10.1111/j.1556-4029.2007.00627.x

[55] Rosas, A. & Bastir, M. Thin-plate spline analysis of allometry and sexual dimorphism in the human craniofacial complex. *Am. J. Phys. Anthropol.* **117**, 236–245. 10.1002/ajpa.10023 (2002). 11842403 10.1002/ajpa.10023

[56] Gonzalez, P. N., Bernal, V. & Perez, S. I. Analysis of sexual dimorphism of craniofacial traits using geometric morphometric techniques. *Int. J. Osteoarchaeol.* **21**, 82–91. 10.1002/oa.1109 (2011).

[57] Del Bove, A., Profico, A., Riga, A., Bucchi, A. & Lorenzo, C. A geometric morphometric approach to the study of sexual dimorphism in the modern human frontal bone. *Am. J. Phys. Anthropol.* **173**, 643–654. 10.1002/ajpa.24154 (2020). 33025582 10.1002/ajpa.24154

[58] Milella, M., Franklin, D., Belcastro, M. G. & Cardini, A. Sexual differences in human cranial morphology: Is one sex more variable or one region more dimorphic?. *Anatom. Record* **304**, 2789–2810. 10.1002/ar.24626 (2021). 10.1002/ar.24626 33773067

[59] Del Bove, A. et al. Mapping sexual dimorphism signal in the human cranium. *Sci. Rep.* **13**, 16847. 10.1038/s41598-023-43007-y (2023). 37803023 10.1038/s41598-023-43007-y PMC10558540

[60] Bertsatos, A., Chovalopoulou, M.-E., Brůžek, J. & Bejdová, Š. Advanced procedures for skull sex estimation using sexually dimorphic morphometric features. *Int. J. Legal Med.*10.1007/s00414-020-02334-9 (2020). 32504147 10.1007/s00414-020-02334-9

[61] Toneva, D. et al. Machine learning approaches for sex estimation using cranial measurements. *Int. J. Legal Med.* **135**, 951–966. 10.1007/s00414-020-02460-4 (2021). 33179173 10.1007/s00414-020-02460-4

[62] d’Oliveira Coelho, J. & Curate, F. CADOES: An interactive machine-learning approach for sex estimation with the pelvis. *Forensic Sci. Int.*10.1016/j.forsciint.2019.109873 (2019). 31382223 10.1016/j.forsciint.2019.109873

[63] Constantinou, C. & Nikita, E. SexEst: An open access web application for metric skeletal sex estimation. 10.1002/oa.3109

[64] Mahfouz, M. et al. Patella sex determination by 3D statistical shape models and nonlinear classifiers. *Forensic Sci. Int.* **173**, 161–170. 10.1016/j.forsciint.2007.02.024 (2007). 17482786 10.1016/j.forsciint.2007.02.024

[65] Curate, F. et al. A method for sex estimation using the proximal femur. *Forensic Sci. Int.* **266**, 579.e571-579.e577. 10.1016/j.forsciint.2016.06.011 (2016). 10.1016/j.forsciint.2016.06.011 27373600

[66] Curate, F. et al. Sex determination from the femur in Portuguese populations with classical and machine-learning classifiers. *J. Forensic Legal Med.* **52**, 75–81. 10.1016/j.jflm.2017.08.011 (2017). 10.1016/j.jflm.2017.08.011 28866285

[67] du Jardin, P., Ponsaillé, J., Alunni-Perret, V. & Quatrehomme, G. A comparison between neural network and other metric methods to determine sex from the upper femur in a modern French population. *Forensic Sci. Int.* **192**, 127e.121-127e.126. 10.1016/j.forsciint.2009.07.014 (2009). 10.1016/j.forsciint.2009.07.014 19733989

[68] Bertsatos, A., Garoufi, N. & Chovalopoulou, M.-E. Advancements in sex estimation using the diaphyseal cross-sectional geometric properties of the lower and upper limbs. *Int. J. Legal Med.*10.1007/s00414-020-02437-3 (2020). 33029676 10.1007/s00414-020-02437-3

[69] Imaizumi, K. et al. Development of a sex estimation method for skulls using machine learning on three-dimensional shapes of skulls and skull parts. *Forensic Imaging* **22**, 200393. 10.1016/j.fri.2020.200393 (2020).

[70] Godinho, R. M., Umbelino, C. & Gonçalves, C. Mesolithic and chalcolithic mandibular morphology: using geometric morphometrics to reconstruct incomplete specimens and analyse morphology. *Open Archaeol.* **8**, 536–549. 10.1515/opar-2022-0247 (2022).

[71] Godinho, R. M. et al. Mandibular morphology and the Mesolithic-Neolithic transition in Westernmost Iberia. *Sci. Rep.* **13**, 16648. 10.1038/s41598-023-42846-z (2023). 37789074 10.1038/s41598-023-42846-z PMC10547775

[72] Katz, D. C., Grote, M. N. & Weaver, T. D. Changes in human skull morphology across the agricultural transition are consistent with softer diets in preindustrial farming groups. *Proc. Natl. Acad. Sci.*10.1073/pnas.1702586114 (2017). 28739900 10.1073/pnas.1702586114 PMC5576786

[73] von Cramon-Taubadel, N. Global human mandibular variation reflects differences in agricultural and hunter-gatherer subsistence strategies. *Proc. Natl. Acad. Sci.* **108**, 19546–19551. 10.1073/pnas.1113050108 (2011). 22106280 10.1073/pnas.1113050108 PMC3241821

[74] Galland, M., Van Gerven, D. P., Von Cramon-Taubadel, N. & Pinhasi, R. 11,000 years of craniofacial and mandibular variation in Lower Nubia. *Sci. Rep.* **6**, 31040. 10.1038/srep31040 (2016). 27503560 10.1038/srep31040 PMC4977491

[75] Pokhojaev, A., Avni, H., Sella-Tunis, T., Sarig, R. & May, H. Changes in human mandibular shape during the Terminal Pleistocene-Holocene Levant. *Sci. Rep.* **9**, 8799. 10.1038/s41598-019-45279-9 (2019). 31217474 10.1038/s41598-019-45279-9 PMC6584575

[76] Crevecoeur, I., Dias-Meirinho, M.-H., Zazzo, A., Antoine, D. & Bon, F. New insights on interpersonal violence in the Late Pleistocene based on the Nile valley cemetery of Jebel Sahaba. *Sci. Rep.* **11**, 9991. 10.1038/s41598-021-89386-y (2021). 34045477 10.1038/s41598-021-89386-y PMC8159958

[77] Balci, Y., Yavuz, M. F. & Cağdir, S. Predictive accuracy of sexing the mandible by ramus flexure. *Homo* **55**, 229–237. 10.1016/j.jchb.2004.07.006 (2005). 15803768 10.1016/j.jchb.2004.07.006

[78] Donnelly, S. M., Hens, S. M., Rogers, N. L. & Schneider, K. L. Technical note: A blind test of mandibular ramus flexure as a morphologic indicator of sexual dimorphism in the human skeleton. *Am. J. Phys. Anthropol.* **107**, 363–366 (1998). 9821499 10.1002/(SICI)1096-8644(199811)107:3<363::AID-AJPA11>3.0.CO;2-Y

[79] Franklin, D., O’Higgins, P. & Oxnard, C. E. Sexual dimorphism in the mandible of indigenous South Africans: A geometric morphometric approach. *S. Afr. J. Sci.* **104**, 101–106 (2008).

[80] Franklin, D., O’Higgins, P., Oxnard, C. E. & Dadour, I. Discriminant function sexing of the mandible of Indigenous South Africans. *Forensic Sci. Int.* **179**, 84.e81-84.e85. 10.1016/j.forsciint.2008.03.014 (2008). 10.1016/j.forsciint.2008.03.014 18467049

[81] Giles, E. Sex determination by discriminant function analysis of the mandible. *Am. J. Phys. Anthropol.* **22**, 129–135. 10.1002/ajpa.1330220212 (1964). 14243698 10.1002/ajpa.1330220212

[82] Hanihara, K. Sex diagnosis of japanese skulls and scapulae by. *J. Anthropol. Soc. Nippon* **67**, 191–197. 10.1537/ase1911.67.191 (1959).

[83] Haun, S. J. Brief communication: A study of the predictive accuracy of mandibular ramus flexure as a singular morphologic indicator of sex in an archaeological sample. *Am. J. Phys. Anthropol.* **111**, 429–432. 10.1002/(sici)1096-8644(200003)111:3%3c429::aid-ajpa9%3e3.0.co;2-1 (2000). 10685041 10.1002/(SICI)1096-8644(200003)111:3<429::AID-AJPA9>3.0.CO;2-1

[84] Hill, C. A. Technical note: evaluating mandibular ramus flexure as a morphological indicator of sex. *Am. J. Phys. Anthropol.* **111**, 573–577 (2000). 10727974 10.1002/(SICI)1096-8644(200004)111:4<573::AID-AJPA11>3.0.CO;2-I

[85] Kemkes-Grottenthaler, A., Löbig, F. & Stock, F. Mandibular ramus flexure and gonial eversion as morphologic indicators of sex. *Homo* **53**, 97–111. 10.1078/0018-442X-00039 (2002). 12489410 10.1078/0018-442x-00039

[86] Kuha, A., Ackermann, J., Junno, J. A., Oettlé, A. & Oura, P. Deep learning in sex estimation from photographed human mandible using the human osteological research collection. *Legal Med.* **70**, 102476. 10.1016/j.legalmed.2024.102476 (2024). 38964075 10.1016/j.legalmed.2024.102476

[87] Loth, S. R. & Henneberg, M. Mandibular ramus flexure: A new morphologic indicator of sexual dimorphism in the human skeleton. *Am. J. Phys. Anthropol. Off. Publ. Am. Assoc. Phys. Anthropol.* **99**, 473–485 (1996). 10.1002/(SICI)1096-8644(199603)99:3<473::AID-AJPA8>3.0.CO;2-X 8850186

[88] Loth, S. R. & Henneberg, M. Mandibular ramus flexure is a good indicator of sexual dimorphism. *Am. J. Phys. Anthropol.* **105**, 91–92 (1998). 9537932 10.1002/(SICI)1096-8644(199801)105:1<91::AID-AJPA9>3.0.CO;2-G

[89] Loth, S. R. & Henneberg, M. Sexually dimorphic mandibular morphology in the first few years of life. *Am. J. Phys. Anthropol.* **115**, 179–186. 10.1002/ajpa.1067 (2001). 11385604 10.1002/ajpa.1067

[90] Oettlé, A. C., Pretorius, E. & Steyn, M. Geometric morphometric analysis of mandibular ramus flexure. *Am. J. Phys. Anthropol. Off. Publ. Am. Assoc. Phys. Anthropol.* **128**, 623–629. 10.1002/ajpa.20207 (2005). 10.1002/ajpa.20207 15861427

[91] Oettlé, A. C., Pretorius, E. & Steyn, M. Geometric morphometric analysis of the use of mandibular gonial eversion in sex determination. *Homo* **60**, 29–43. 10.1016/j.jchb.2007.01.003 (2009). 18996521 10.1016/j.jchb.2007.01.003

[92] Ogawa, Y., Imaizumi, K., Miyasaka, S. & Yoshino, M. Discriminant functions for sex estimation of modern Japanese skulls. *J. Forensic Legal Med.* **20**, 234–238. 10.1016/j.jflm.2012.09.023 (2013). 10.1016/j.jflm.2012.09.023 23622466

[93] Plotkin, L. I., Bruzzaniti, A. & Pianeta, R. Sexual dimorphism in the musculoskeletal system: Sex hormones and beyond. *J. Endocr. Soc.*10.1210/jendso/bvae153 (2024). 39309123 10.1210/jendso/bvae153 PMC11413583

[94] Pretorius, E., Steyn, M. & Scholtz, Y. Investigation into the usability of geometric morphometric analysis in assessment of sexual dimorphism. *Am. J. Phys. Anthropol.* **129**, 64–70. 10.1002/ajpa.20251 (2006). 16245344 10.1002/ajpa.20251

[95] Rmoutilová, R. et al. Classification performance of the Sella-Tunis et al. (2017) sex estimation method in Czech population: different posterior probability threshold approaches. *Int. J. Legal Med.*10.1007/s00414-024-03241-z (2024). 38714567 10.1007/s00414-024-03241-z

[96] Schmittbuhl, M., Le Minor, J. M., Schaaf, A. & Mangin, P. The human mandible in lateral view: elliptical fourier descriptors of the outline and their morphological analysis. *Ann. Anat. Anatomischer. Anzeiger.* **184**, 199–207. 10.1016/S0940-9602(02)80021-8 (2002). 11936202 10.1016/S0940-9602(02)80021-8

[97] Steyn, M. & İşcan, M. Y. Sexual dimorphism in the crania and mandibles of South African whites. *Forensic Sci. Int.* **98**, 9–16. 10.1016/S0379-0738(98)00120-0 (1998). 10036755 10.1016/s0379-0738(98)00120-0

[98] Toneva, D. H., Nikolova, S. Y., Fileva, N. F. & Zlatareva, D. K. Size and shape of human mandible: Sex differences and influence of age on sex estimation accuracy. *Legal Med.* **65**, 102322. 10.1016/j.legalmed.2023.102322 (2023). 37722156 10.1016/j.legalmed.2023.102322

[99] Murail, P., Bruzek, J. & Braga, J. A new approach to sexual diagnosis in past populations. Practical adjustments from Van Vark’s procedure. *Int. J. Osteoarchaeol.* **9**, 39–53 (1999).

[100] Albanese, J., Cardoso, H. F. V. & Saunders, S. R. Universal methodology for developing univariate sample-specific sex determination methods: an example using the epicondylar breadth of the humerus. *J. Archaeol. Sci.* **32**, 143–152. 10.1016/j.jas.2004.08.003 (2005).

[101] Garcia, S. Is the circumference at the nutrient foramen of the tibia of value to sex determination on human osteological collections? Testing a new method. *Int. J. Osteoarchaeol.* **22**, 361–365. 10.1002/oa.1202 (2012).

[102] Gonçalves, D., Granja, R., Cardoso, F. A. & de Carvalho, A. F. Sample-specific sex estimation in archaeological contexts with commingled human remains: a case study from the Middle Neolithic cave of Bom Santo in Portugal. *J. Archaeol. Sci.* **49**, 185–191. 10.1016/j.jas.2014.05.011 (2014).

[103] İşcan, M. Y., Loth, S. R., King, C. A., Shihai, D. & Yoshino, M. Sexual dimorphism in the humerus: A comparative analysis of Chinese. *Jpn. Thais. Forensic Sci. Int.* **98**, 17–29. 10.1016/S0379-0738(98)00119-4 (1998). 10.1016/s0379-0738(98)00119-4 10036756

[104] Bidmos, M. A. & Mazengenya, P. Accuracies of discriminant function equations for sex estimation using long bones of upper extremities. *Int. J. Legal Med.* **135**, 1095–1102. 10.1007/s00414-020-02458-y (2021). 33179172 10.1007/s00414-020-02458-y

[105] Greene, D. L., Ewing, G. H. & Armelagos, G. J. Dentition of a mesolithic population from Wadi Halfa, Sudan. *Am. J. Phys. Anthropol.* **27**, 41–55. 10.1002/ajpa.1330270107 (1967). 6049819 10.1002/ajpa.1330270107

[106] Irish, J. D. Population continuity vs. discontinuity revisited: Dental affinities among late Paleolithic through Christian-era Nubians. *Am. J. Phys. Anthropol.* **128**, 520–535. 10.1002/ajpa.20109 (2005). 15895433 10.1002/ajpa.20109

[107] Crevecoeur, I., Rougier, H., Grine, F. & Froment, A. Modern human cranial diversity in the late pleistocene of Africa and Eurasia: Evidence from Nazlet Khater. *Peştera cu Oase, Hofmeyr.* **140**, 347–358. 10.1002/ajpa.21080 (2009). 10.1002/ajpa.21080 19425102

[108] Martin, N. et al. From hunter-gatherers to food producers: New dental insights into the Nile Valley population history (Late Paleolithic–Neolithic). *Am. J. Biol. Anthropol.* **184**, e24948. 10.1002/ajpa.24948 (2024). 38733278 10.1002/ajpa.24948

[109] Anderson, J. E. *The Prehistory of Nubia* (Fort Burgwin Research Center and Southern Methodist University Press, 1968).

[110] Pagani, L. & Crevecoeur, I. *Modern Human Origins and Dispersal, Words, Bones, Genes, Tools* (Kerns Verlag (DFG Center for Advanced Studies Series), UK, 2019).

[111] Crevecoeur, I., Matu, M., Dias-Meirinho, M.-H., Bayle, P. & Pearson, O. *Du Big dry à l’Holocène en Afrique de l’est et au-delà* (Séances de la Société Préhistorique Française, 2023).

[112] Hisham, S., Zainun, K. A. & Ibrahim, M. A. Observer error in assessing age-related morphology using digital photographs of the pubic symphyseal face. *Canadian Soc. Forensic Sci. J.* **55**, 213–220. 10.1080/00085030.2022.2053279 (2022).

[113] Villotte, S., Kacki, S. & Thomas, A. Sex estimation of the human os coxae in archeological contexts: An advocacy of using both diagnose sexuelle probabiliste and Brůžek’s morphoscopic method. *Int. J. Osteoarchaeol.* **34**, e3334. 10.1002/oa.3334 (2024).

[114] Pilloud, M. A., Adams, D. M. & Hefner, J. T. Observer error and its impact on ancestry estimation using dental morphology. *Int. J. Legal Med.* **133**, 949–962. 10.1007/s00414-018-1985-3 (2019). 30564914 10.1007/s00414-018-1985-3

[115] Williams, B. A. & Rogers, T. L. Evaluating the accuracy and precision of cranial morphological traits for sex determination. *J. Forensic Sci.* **51**, 729–735. 10.1111/j.1556-4029.2006.00177.x (2006). 16882212 10.1111/j.1556-4029.2006.00177.x

[116] Kemkes, A. & Göbel, T. Metric assessment of the “mastoid triangle” for sex determination: A validation study. *J. Forensic Sci.* **51**, 985–989. 10.1111/j.1556-4029.2006.00232.x (2006). 17018073 10.1111/j.1556-4029.2006.00232.x

[117] Godde, K., Thompson, M. M. & Hens, S. M. Sex estimation from cranial morphological traits: use of the methods across American Indians, modern North Americans, and ancient Egyptians. *Homo-J. Comparat. Human Biol.*10.1016/j.jchb.2018.09.003 (2018). 10.1016/j.jchb.2018.09.003 30269926

[118] Smith, H. F. Which cranial regions reflect molecular distances reliably in humans? Evidence from three-dimensional morphology. *Am. J. Human Biol.* **21**, 36–47. 10.1002/ajhb.20805 (2009). 18663742 10.1002/ajhb.20805

[119] Harvati, K. & Weaver, T. D. Human cranial anatomy and the differential preservation of population history and climate signatures. *Anat. Rec. A Discov. Mol. Cell. Evol. Biol.* **288**, 1225–1233 (2006). 17075844 10.1002/ar.a.20395

[120] Roseman, C. C. Detecting interregionally diversifying natural selection on modern human cranial form by using matched molecular and morphometric data. *P. Natl. Acad. Sci. USA* **101**, 12824–12829. 10.1073/pnas.0402637101 (2004). 10.1073/pnas.0402637101 PMC516480 15326305

[121] Roseman, C. C. & Weaver, T. D. Multivariate apportionment of global human craniometric diversity. *Am. J. Phys. Anthropol.* **125**, 257–263. 10.1002/ajpa.10424 (2004). 15386236 10.1002/ajpa.10424

[122] González-José, R. et al. Functional-cranial approach to the influence of economic strategy on skull morphology. *Am. J. Phys. Anthropol.* **128**, 757–771. 10.1002/ajpa.20161 (2005). 16028224 10.1002/ajpa.20161

[123] Godde, K. Secular trends in cranial morphological traits: a socioeconomic perspective of change and sexual dimorphism in North Americans 1849–1960. *Ann. Hum. Biol.* **42**, 255–261. 10.3109/03014460.2014.941399 (2015). 10.3109/03014460.2014.941399 25156659

[124] Weiss, K. M. On the systematic bias in skeletal sexing. *Am. J. Phys. Anthropol.* **37**, 239–249. 10.1002/ajpa.1330370208 (1972). 5085497 10.1002/ajpa.1330370208

[125] Cintas-Peña, M. & Herrero-Corral, A. M. Missing prehistoric women? Sex ratio as an indicator for analyzing the population of Iberia from the 8th to the 3rd millennia B.C. *Archaeol. Anthropol. Sci.* **12**, 263. 10.1007/s12520-020-01215-x (2020).

[126] Cardoso, H. F. V. Brief communication: The collection of identified human skeletons housed at the Bocage Museum (National Museum of Natural History), Lisbon Portugal. *Am. J. Phys. Anthropol.* **129**, 173–176. 10.1002/ajpa.20228 (2006). 16323180 10.1002/ajpa.20228

[127] Coquerelle, M. et al. Sexual dimorphism of the human mandible and its association with dental development. *Am. J. Phys. Anthropol.* **145**, 192–202. 10.1002/Ajpa.21485 (2011). 21365613 10.1002/ajpa.21485

[128] AlQahtani, S. J., Hector, M. P. & Liversidge, H. M. Brief communication: The London atlas of human tooth development and eruption. *Am. J. Phys. Anthropol.* **142**, 481–490. 10.1002/ajpa.21258 (2010). 20310064 10.1002/ajpa.21258

[129] Bruzek, J. A method for visual determination of sex, using the human hip bone. *Am. J. Phys. Anthropol.* **117**, 157–168. 10.1002/ajpa.10012 (2002). 11815949 10.1002/ajpa.10012

[130] Murail, P., Bruzek, J., Houët, F. & Cunha, E. DSP: a tool for probabilistic sex diagnosis using worldwide variability in hip-bone measurements. *Bull. et Mémoires de la Société d’Anthropol. de Paris* **17**, 167–176 (2005).

[131] Fedorov, A. et al. 3D slicer as an image computing platform for the quantitative imaging network. *Magn. Reson. Imaging* **30**, 1323–1341. 10.1016/j.mri.2012.05.001 (2012). 22770690 10.1016/j.mri.2012.05.001 PMC3466397

[132] Geomorph: Software for geometric morphometric analyses. R package version 4.0.6 (2023).

[133] Schlager, S., Jefferis, G., Ian, D. & Schlager, M. S. (Academic Press, 2023).

[134] Godinho, R. M., O’Higgins, P. & Gonçalves, C. Assessing the reliability of virtual reconstruction of mandibles. *Am. J. Phys. Anthropol.* **172**, 723–734. 10.1002/ajpa.24095 (2020). 32557582 10.1002/ajpa.24095

[135] Gunz, P., Mitteroecker, P., Neubauer, S., Weber, G. W. & Bookstein, F. L. Principles for the virtual reconstruction of hominin crania. *J. Hum. Evol.* **57**, 48–62. 10.1016/j.jhevol.2009.04.004 (2009). 19482335 10.1016/j.jhevol.2009.04.004

[136] Neeser, R., Ackermann, R. R. & Gain, J. Comparing the accuracy and precision of three techniques used for estimating missing landmarks when reconstructing fossil hominin crania. *Am. J. Phys. Anthropol.* **140**, 1–18. 10.1002/ajpa.21023 (2009). 19208416 10.1002/ajpa.21023

[137] Senck, S., Bookstein, F. L., Benazzi, S., Kastner, J. & Weber, G. W. Virtual reconstruction of modern and fossil hominoid crania: consequences of reference sample choice. *Anat. Rec.* **298**, 827–841. 10.1002/ar.23104 (2015). 10.1002/ar.23104 25689596

[138] Mitteroecker, P., Gunz, P., Windhager, S. & Schaefer, K. A brief review of shape, form, and allometry in geometric morphometrics, with applications to human facial morphology. *Hystrix-Italian J. Mammal.* **24**, 59–66. 10.4404/hystrix-24.1-6369 (2013).

[139] Zelditch, M. L., Swiderski, D. L., Sheets, H. D. & Fink, W. L. *Geometric Morphometrics For Biologists: A Primer* (Elsevier, 2012).

[140] O’Higgins, P. The study of morphological variation in the hominid fossil record: biology, landmarks and geometry. *J. Anat.* **197**, 103–120. 10.1046/j.1469-7580.2000.19710103.x (2000). 10999273 10.1046/j.1469-7580.2000.19710103.x PMC1468110

[141] Klingenberg, C. P. Size, shape, and form: concepts of allometry in geometric morphometrics. *Dev. Genes. Evol.* **226**, 113–137. 10.1007/s00427-016-0539-2 (2016). 27038023 10.1007/s00427-016-0539-2 PMC4896994

[142] Hammer, O. *PAST reference manual 4.12*. (2022).

[143] Courtenay, L. A., Aramendi, J. & González-Aguilera, D. A graph-based mathematical model for more efficient dimensionality reduction of landmark data in geometric morphometrics. *Evol. Biol.*10.1007/s11692-024-09636-5 (2024).

[144] Abellán, N., Baquedano, E. & Domínguez-Rodrigo, M. High-accuracy in the classification of butchery cut marks and crocodile tooth marks using machine learning methods and computer vision algorithms. *Geobios* **72–73**, 12–21. 10.1016/j.geobios.2022.07.001 (2022).

[145] Moclán, A. et al. Identifying the bone-breaker at the Navalmaíllo Rock Shelter (Pinilla del Valle, Madrid) using machine learning algorithms. *Archaeol. Anthropol. Sci.* **12**, 46. 10.1007/s12520-020-01017-1 (2020).

[146] Courtenay, L. A. & González-Aguilera, D. Geometric morphometric data augmentation using generative computational learning algorithms. *Appl. Sci.* **10**, 9133 (2020).

[147] McPherron, S. P., Archer, W., Otárola-Castillo, E. R., Torquato, M. G. & Keevil, T. L. Machine learning, bootstrapping, null models, and why we are still not 100% sure which bone surface modifications were made by crocodiles. *J. Human Evol.* **164**, 103071. 10.1016/j.jhevol.2021.103071 (2022). 34635347 10.1016/j.jhevol.2021.103071

[148] Kuhn, M. *et al.* Caret: Classification and Regression Training. (2024).

[149] Deane-Mayer, Z. A., Knowles, J. E. & López, A. Package ‘caretEnsemble’. (2024).

[150] Lantz, B. *Machine learning with R* (Packt Publishing, 2013).

[151] Domínguez-Rodrigo, M. Successful classification of experimental bone surface modifications (BSM) through machine learning algorithms: a solution to the controversial use of BSM in paleoanthropology?. *Archaeol. Anthropol. Sci.* **11**, 2711–2725. 10.1007/s12520-018-0684-9 (2019).

