# Correction: Coupling geometric morphometrics and machine learning for mandibular sex estimation in Late Pleistocene and Late Modern populations

**DOI:** 10.1038/s41598-026-40435-4

**Published:** 2026-02-17

**Authors:** Ricardo Miguel Godinho, Isabelle Crevecoeur, Susana Garcia, Rebecca Whiting, Julia Aramendi

**Affiliations:** 1 Interdisciplinary Center for Archaeology and Evolution of Human Behaviour (ICArEHB), Faculdade das Ciências Humanas E Sociais, University of Algarve, Universidade Do Algarve, Campus Gambelas, 8005-139 Faro, Portugal; 2 UMR 5199‑PACEA, CNRS, Université de Bordeaux, B8, Allée Geoffroy Saint‑Hilaire, CS 50023, 33615 Pessac Cedex, France; 3 Centre for Public Administration and Public Policies, Institute of Social and Political Sciences, MUHNAC, Universidade de Lisboa, Rua Almerindo Lessa, 1300-663 Lisbon, Portugal; 4 The British Museum, London, UK; 5 McDonald Institute for Archaeological Research, University of Cambridge, Cambridge, CB2 1TN UK

Correction to: *Scientific Reports* 10.1038/s41598-025-31365-8, published online 19 December 2025

The original version of this Article contained an error in the name of author Isabelle Crevecoeur, which was incorrectly given as Isabelle Crevecouer.

The original Article has been corrected.

