# Supplementary material for: Coupling geometric morphometrics and machine learning for mandibular sex estimation in Late Pleistocene and Late Modern populations
Source: Sci Rep. 2025 Dec 19;16:1775. doi: 10.1038/s41598-025-31365-8 (PMC12804928; doi:10.1038/s41598-025-31365-8)
Supplement: Supplementary file 1 — Supplementary Information. [file 41598_2025_31365_MOESM1_ESM.docx]

**Coupling Geometric Morphometrics and Machine Learning in mandibular sex estimation: testing Late Pleistocene and Late Modern populations**

# Authors

Ricardo Miguel **Godinho**^1^

Corresponding author

email: rmgodinho@ualg.pt

Phone number: +351963281822

ORCID: 0000-0003-0107-9577

Affiliation:

1) Interdisciplinary Center for Archaeology and Evolution of Human Behaviour (ICArEHB), University of Algarve, Faculdade das Ciências Humanas e Sociais, Universidade do Algarve, Campus Gambelas, 8005-139, Faro, Portugal

Isabelle **Crevecouer**^2^

2) UMR 5199‑PACEA, CNRS, Université de Bordeaux, B8, Allée Geoffroy Saint‑Hilaire, CS 50023, 33615 Pessac Cedex, France.

Susana **Garcia**^3^

3) Centre for Public Administration and Public Policies, Institute of Social and Political Sciences, MUHNAC, Universidade de Lisboa, Rua Almerindo Lessa, 1300-663

Lisbon, Portugal

Rebecca **Whiting**^4^

4) Department of Egypt and Sudan, The British Museum, London, UK

Julia **Aramendi**^5^

5) McDonald Institute for Archaeological Research, University of Cambridge, CB2 1TN, UK

# Supplementary information


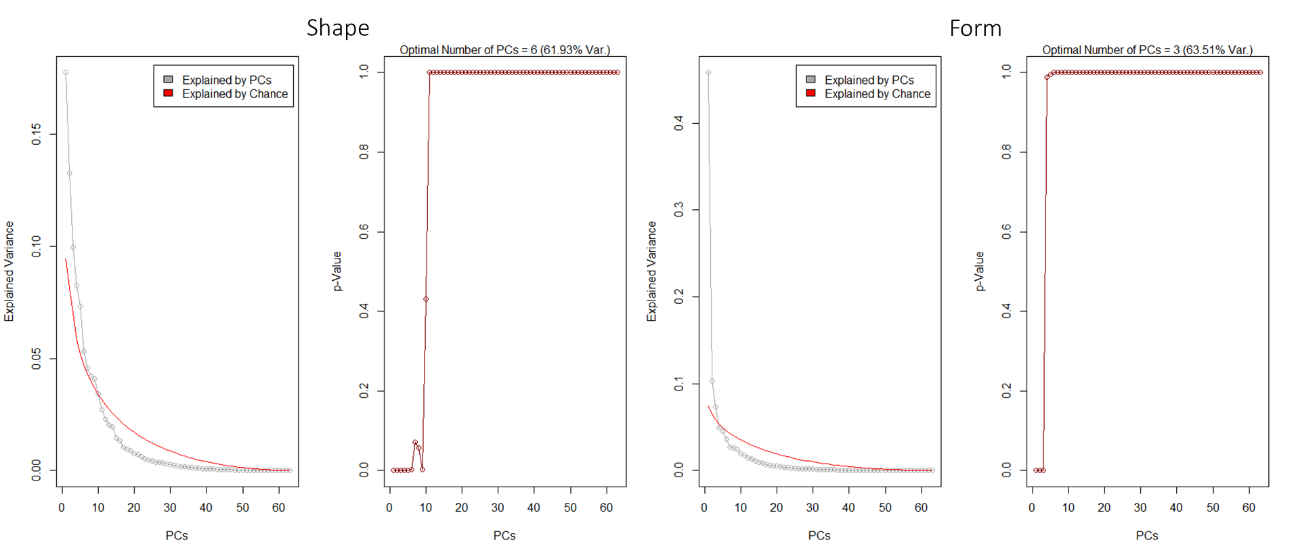


Figure SI 1: *p*-values for the significance of each PC in shape and form space, alongside the proportion of accumulated variance for each case.


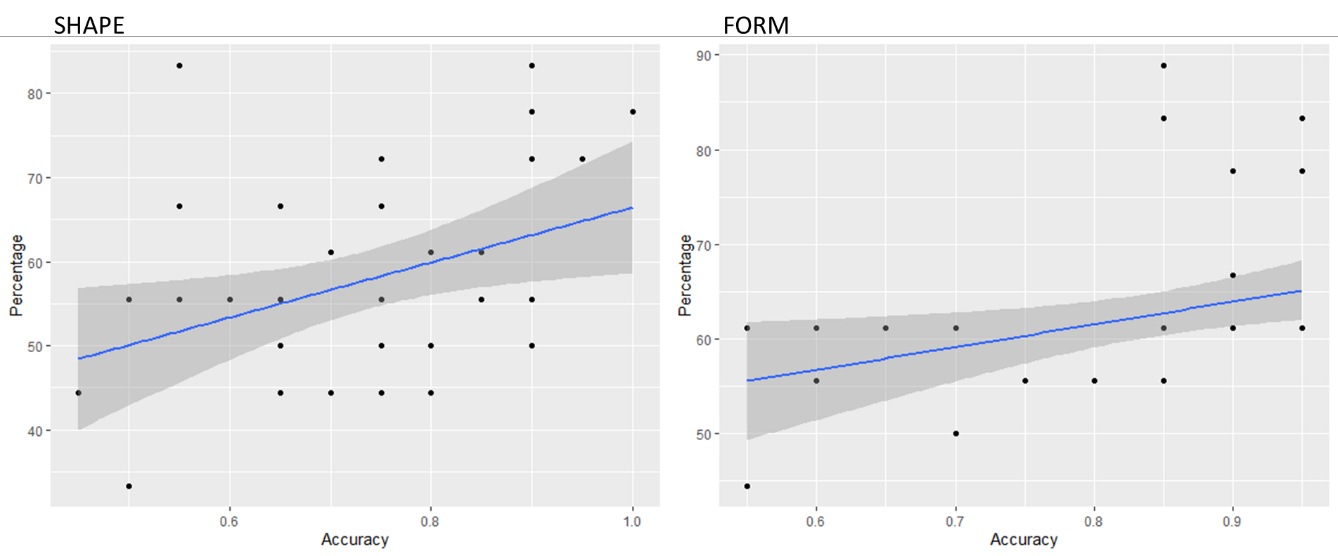


Figure SI 2: Linear regression of model accuracy trained on modern data as a predictor of model accuracy on archaeological data, using shape (R2 = 0.126, F = 0.018) and form (R2 = 0.125, F = 0.019) variables. See Table SI 17 for more details.

Table SI 1: PERMANOVA results comparing different groups in shape and form space. Upper triangles show F values and lower triangles P values (significantly different P values highlighted in orange).

| **Shape space**; upper triangle: F values; lower triangle: Bonferroni adjusted p values | | |  |  |
| --- | --- | --- | --- | --- |
|  | Late modern, Portugal, Male | Late modern, Portugal, Female | Late stone age, Sudan, Male | Late stone age, Sudan, Female |
| Late modern, Portugal, Male | 0 | 2,633 | 16,5 | 14,49 |
| Late modern, Portugal, Female | 0,012 | 0 | 14,02 | 11,16 |
| Late stone age, Sudan, Male | 0,0006 | 0,0006 | 0 | 1,434 |
| Late stone age, Sudan, Female | 0,0006 | 0,0006 | 0,864 | 0 |
|  |  |  |  |  |
| **Form space**; upper triangle: F values; lower triangle: Bonferroni adjusted p values | | |  |  |
|  | Late modern, Portugal, Male | Late modern, Portugal, Female | Late stone age, Sudan, Male | Late stone age, Sudan, Female |
| Late modern, Portugal, Male | 0 | 20,46 | 17,86 | 9,601 |
| Late modern, Portugal, Female | 0,0006 | 0 | 42,66 | 16,35 |
| Late stone age, Sudan, Male | 0,0006 | 0,0006 | 0 | 6,117 |
| Late stone age, Sudan, Female | 0,0006 | 0,0006 | 0,0222 | 0 |

Table SI 2: Regression of shape and form PC scores against centroid size in the late modern Luis Lopes (Portugal) and Late Pleistocene Jebel Sahaba (Sudan) samples. Statistically significant relationships are highlighted in bold and orange background.

| **Shape space** | full.sample.rsquared | full.sample.adj.rsquared | full.sample.F.statistic | full.sample.p.value | Portugal.rsquared | Portugal.adj.rsquared | Portugal.F.statistic | Portugal.p.value | Sudan.rsquared | Sudan.adj.rsquared | Sudan.F.statistic | Sudan.p.value |
| --- | --- | --- | --- | --- | --- | --- | --- | --- | --- | --- | --- | --- |
| PC 1 | 0,1782 | 0,1683 | 17,9988 | **0,0001** | 0,0029 | -0,0125 | 0,1859 | 0,6678 | 0,0069 | -0,0552 | 0,1109 | 0,7435 |
| PC 2 | 0,0023 | -0,0097 | 0,1902 | 0,6639 | 0,0000 | -0,0154 | 0,0003 | 0,9853 | 0,0001 | -0,0624 | 0,0008 | 0,9771 |
| PC 3 | 0,1187 | 0,1080 | 11,1740 | **0,0012** | 0,1126 | 0,0989 | 8,2449 | **0,0055** | 0,1574 | 0,1047 | 2,9881 | 0,1031 |
| PC 4 | 0,1455 | 0,1352 | 14,1293 | **0,0003** | 0,3027 | 0,2920 | 28,2148 | **0,0000** | 0,2351 | 0,1873 | 4,9178 | **0,0414** |
| PC 5 | 0,0232 | 0,0114 | 1,9692 | 0,1643 | 0,0487 | 0,0341 | 3,3271 | 0,0727 | 0,0113 | -0,0505 | 0,1828 | 0,6747 |
| PC 6 | 0,0015 | -0,0105 | 0,1266 | 0,7229 | 0,0110 | -0,0042 | 0,7237 | 0,3980 | 0,1488 | 0,0956 | 2,7969 | 0,1139 |
| PC 7 | 0,0294 | 0,0177 | 2,5141 | 0,1166 | 0,0039 | -0,0114 | 0,2537 | 0,6162 | 0,4863 | 0,4542 | 15,1462 | **0,0013** |
| PC 8 | 0,0806 | 0,0695 | 7,2771 | **0,0085** | 0,0838 | 0,0697 | 5,9440 | **0,0175** | 0,0585 | -0,0003 | 0,9941 | 0,3336 |
| PC 9 | 0,0013 | -0,0107 | 0,1082 | 0,7430 | 0,0091 | -0,0061 | 0,5969 | 0,4426 | 0,0325 | -0,0280 | 0,5367 | 0,4744 |
| PC 10 | 0,0144 | 0,0025 | 1,2095 | 0,2746 | 0,0381 | 0,0233 | 2,5779 | 0,1132 | 0,0124 | -0,0494 | 0,2001 | 0,6607 |
| PC 11 | 0,0146 | 0,0027 | 1,2309 | 0,2704 | 0,0129 | -0,0023 | 0,8489 | 0,3603 | 0,0238 | -0,0372 | 0,3895 | 0,5413 |
| PC 12 | 0,0090 | -0,0029 | 0,7537 | 0,3878 | 0,0010 | -0,0143 | 0,0667 | 0,7970 | 0,0382 | -0,0219 | 0,6360 | 0,4368 |
| PC 13 | 0,0544 | 0,0430 | 4,7778 | **0,0316** | 0,0249 | 0,0099 | 1,6618 | 0,2019 | 0,1897 | 0,1390 | 3,7450 | 0,0708 |
| PC 14 | 0,0082 | -0,0037 | 0,6889 | 0,4089 | 0,0049 | -0,0105 | 0,3169 | 0,5754 | 0,0104 | -0,0515 | 0,1679 | 0,6874 |
| PC 15 | 0,0034 | -0,0086 | 0,2813 | 0,5973 | 0,0006 | -0,0148 | 0,0360 | 0,8501 | 0,1358 | 0,0818 | 2,5151 | 0,1323 |
| PC 16 | 0,0001 | -0,0120 | 0,0048 | 0,9449 | 0,0048 | -0,0105 | 0,3133 | 0,5776 | 0,0852 | 0,0280 | 1,4893 | 0,2400 |
| PC 17 | 0,0013 | -0,0107 | 0,1085 | 0,7427 | 0,0049 | -0,0104 | 0,3232 | 0,5717 | 0,1363 | 0,0823 | 2,5239 | 0,1317 |
| PC 18 | 0,0115 | -0,0004 | 0,9670 | 0,3283 | 0,0084 | -0,0068 | 0,5529 | 0,4598 | 0,0030 | -0,0593 | 0,0485 | 0,8285 |
| PC 19 | 0,0010 | -0,0110 | 0,0870 | 0,7688 | 0,0043 | -0,0111 | 0,2775 | 0,6001 | 0,0145 | -0,0471 | 0,2358 | 0,6338 |
| PC 20 | 0,0018 | -0,0102 | 0,1480 | 0,7015 | 0,0001 | -0,0152 | 0,0095 | 0,9227 | 0,0142 | -0,0474 | 0,2307 | 0,6375 |
| PC 21 | 0,0171 | 0,0053 | 1,4458 | 0,2326 | 0,0405 | 0,0257 | 2,7414 | 0,1026 | 0,0166 | -0,0448 | 0,2705 | 0,6101 |
| PC 22 | 0,0016 | -0,0104 | 0,1317 | 0,7176 | 0,0147 | -0,0004 | 0,9711 | 0,3281 | 0,0064 | -0,0557 | 0,1023 | 0,7532 |
| PC 23 | 0,0140 | 0,0021 | 1,1751 | 0,2815 | 0,0013 | -0,0140 | 0,0856 | 0,7708 | 0,0480 | -0,0115 | 0,8067 | 0,3824 |
|  |  |  |  |  |  |  |  |  |  |  |  |  |
| **Form space** | full.sample.rsquared | full.sample.adj.rsquared | full.sample.F.statistic | full.sample.p.value | Portugal.rsquared | Portugal.adj.rsquared | Portugal.F.statistic | Portugal.p.value | Sudan.rsquared | Sudan.adj.rsquared | Sudan.F.statistic | Sudan.p.value |
| PC 1 | 0,9700 | 0,9696 | 2680,0876 | **0,0000** | 0,9787 | 0,9784 | 2987,7715 | **0,0000** | 0,9863 | 0,9855 | 1155,3539 | **0,0000** |
| PC 2 | 0,0261 | 0,0143 | 2,2202 | 0,1400 | 0,4324 | 0,4237 | 49,5250 | **0,0000** | 0,6287 | 0,6054 | 27,0865 | **0,0001** |
| PC 3 | 0,0003 | -0,0118 | 0,0214 | 0,8840 | 0,0049 | -0,0104 | 0,3194 | 0,5739 | 0,0102 | -0,0517 | 0,1646 | 0,6903 |
| PC 4 | 0,0001 | -0,0120 | 0,0055 | 0,9411 | 0,0168 | 0,0017 | 1,1103 | 0,2959 | 0,0022 | -0,0602 | 0,0349 | 0,8542 |
| PC 5 | 0,0002 | -0,0119 | 0,0133 | 0,9085 | 0,0001 | -0,0153 | 0,0080 | 0,9289 | 0,0609 | 0,0022 | 1,0377 | 0,3235 |
| PC 6 | 0,0013 | -0,0107 | 0,1084 | 0,7428 | 0,0212 | 0,0061 | 1,4082 | 0,2397 | 0,2767 | 0,2315 | 6,1201 | **0,0249** |
| PC 7 | 0,0000 | -0,0120 | 0,0027 | 0,9588 | 0,0105 | -0,0047 | 0,6893 | 0,4094 | 0,0769 | 0,0192 | 1,3323 | 0,2653 |
| PC 8 | 0,0000 | -0,0120 | 0,0002 | 0,9877 | 0,0082 | -0,0071 | 0,5355 | 0,4669 | 0,3225 | 0,2802 | 7,6179 | **0,0139** |
| PC 9 | 0,0000 | -0,0120 | 0,0001 | 0,9913 | 0,0035 | -0,0118 | 0,2284 | 0,6343 | 0,0521 | -0,0071 | 0,8798 | 0,3622 |
| PC 10 | 0,0001 | -0,0119 | 0,0099 | 0,9209 | 0,0002 | -0,0152 | 0,0126 | 0,9110 | 0,0023 | -0,0600 | 0,0374 | 0,8492 |
| PC 11 | 0,0002 | -0,0118 | 0,0191 | 0,8904 | 0,0080 | -0,0073 | 0,5218 | 0,4727 | 0,0927 | 0,0360 | 1,6353 | 0,2192 |
| PC 12 | 0,0002 | -0,0119 | 0,0141 | 0,9059 | 0,0028 | -0,0126 | 0,1813 | 0,6716 | 0,0095 | -0,0524 | 0,1534 | 0,7005 |
| PC 13 | 0,0001 | -0,0120 | 0,0042 | 0,9485 | 0,0005 | -0,0149 | 0,0312 | 0,8604 | 0,0003 | -0,0622 | 0,0041 | 0,9495 |
| PC 14 | 0,0000 | -0,0120 | 0,0005 | 0,9825 | 0,0001 | -0,0153 | 0,0084 | 0,9271 | 0,0053 | -0,0568 | 0,0858 | 0,7734 |
| PC 15 | 0,0001 | -0,0119 | 0,0115 | 0,9149 | 0,0006 | -0,0147 | 0,0415 | 0,8392 | 0,0611 | 0,0024 | 1,0403 | 0,3229 |
| PC 16 | 0,0000 | -0,0120 | 0,0004 | 0,9846 | 0,0103 | -0,0049 | 0,6785 | 0,4131 | 0,0764 | 0,0187 | 1,3239 | 0,2668 |
| PC 17 | 0,0003 | -0,0118 | 0,0210 | 0,8851 | 0,0002 | -0,0152 | 0,0113 | 0,9157 | 0,0334 | -0,0270 | 0,5529 | 0,4679 |
| PC 18 | 0,0001 | -0,0120 | 0,0067 | 0,9348 | 0,0089 | -0,0063 | 0,5864 | 0,4466 | 0,1391 | 0,0853 | 2,5849 | 0,1274 |

Table SI 3: Results provided by ML algorithms based on PCs accounting for 100% of the total variance in shape and form.

| **Model** | **Data** | **Accuracy** | **Kappa** | **AccLower** | **AccUpper** | **Sensitivity** | **Specificity** | **BalAccuracy** |
| --- | --- | --- | --- | --- | --- | --- | --- | --- |
| **kNN** | shape | 0.75 | 0.5 | 0.509 | 0.9134 | 0.7778 | 0.7273 | 0.7525 |
|  | form | 0.6 | 0.2233 | 0.3605 | 0.8088 | 0.7778 | 0.4545 | 0.6162 |
| **LGR** | shape | 0.8 | 0.596 | 0.5634 | 0.9427 | 0.7778 | 0.8182 | 0.798 |
|  | form | 0.7 | 0.4059 | 0.4572 | 0.8811 | 0.7778 | 0.6364 | 0.7071 |
| **DTC5.0** | shape | 0.7 | 0.3939 | 0.4572 | 0.8811 | 0.6667 | 0.7273 | 0.697 |
|  | form | 0.6 | 0.1398 | 0.3605 | 0.8088 | 0.2222 | 0.9091 | 0.5657 |
| **RF** | shape | 0.7 | 0.3939 | 0.4572 | 0.8811 | 0.6667 | 0.7273 | 0.697 |
|  | form | 0.75 | 0.4898 | 0.509 | 0.9134 | 0.6667 | 0.8182 | 0.7424 |
| **GB** | shape | 0.45 | 0.0577 | 0.2306 | 0.6847 | 0.6667 | 0.2727 | 0.4697 |
|  | form | 0.55 | 0.1176 | 0.3153 | 0.7694 | 0.6667 | 0.4545 | 0.5606 |
| **NB** | shape | 0.55 | 0.1176 | 0.3153 | 0.7694 | 0.6667 | 0.4545 | 0.5606 |
|  | form | 0.7 | 0.3684 | 0.4572 | 0.8811 | 0.4444 | 0.9091 | 0.6768 |
| **LDA** | shape | 0.5 | 0.0309 | 0.272 | 0.728 | 0.3333 | 0.6364 | 0.4848 |
|  | form | 0.7 | 0.4059 | 0.4572 | 0.8811 | 0.7778 | 0.6364 | 0.7071 |
| **PLS** | shape | 0.65 | 0.3 | 0.4078 | 0.8461 | 0.6667 | 0.6364 | 0.6515 |
|  | form | 0.7 | 0.4059 | 0.4572 | 0.8811 | 0.7778 | 0.6364 | 0.7071 |
| **SVMl** | shape | 0.65 | 0.3269 | 0.4078 | 0.8461 | 0.8889 | 0.4545 | 0.6717 |
|  | form | 0.7 | 0.3814 | 0.4572 | 0.8811 | 0.5556 | 0.8182 | 0.6869 |
| **SVMr** | shape | 0.6 | 0.2233 | 0.3605 | 0.8088 | 0.7778 | 0.4545 | 0.6162 |
|  | form | 0.5 | 0.0291 | 0.272 | 0.728 | 0.6667 | 0.3636 | 0.5152 |
| **NNET** | shape | 0.75 | 0.5 | 0.509 | 0.9134 | 0.7778 | 0.7273 | 0.7525 |
|  | form | 0.65 | 0.3 | 0.4078 | 0.8461 | 0.6667 | 0.6364 | 0.6515 |

Table SI 4: Results provided by ML algorithms based on PCs accounting for over 95% of the total variance in shape and form. Algorithms with accuracy above 90% are highlighted in bold.

| **Model** | **Data** | **Accuracy** | **Kappa** | **AccLower** | **AccUpper** | **Sensitivity** | **Specificity** | **BalAccuracy** |
| --- | --- | --- | --- | --- | --- | --- | --- | --- |
| **kNN** | shape | 0.65 | 0.3 | 0.4078 | 0.8461 | 0.6667 | 0.6364 | 0.6515 |
|  | form | **0.9** | **0.802** | **0.683** | **0.9877** | **1** | **0.8182** | **0.9091** |
| **LGR** | shape | 0.8 | 0.604 | 0.5634 | 0.9427 | 0.8889 | 0.7273 | 0.8081 |
|  | form | 0.8 | 0.596 | 0.5634 | 0.9427 | 0.7778 | 0.8182 | 0.798 |
| **DTC5.0** | shape | 0.75 | 0.5 | 0.509 | 0.9134 | 0.7778 | 0.7273 | 0.7525 |
|  | form | **0.9** | **0.802** | **0.683** | **0.9877** | **1** | **0.8182** | **0.9091** |
| **RF** | shape | 0.75 | 0.5 | 0.509 | 0.9134 | 0.7778 | 0.7273 | 0.7525 |
|  | form | **0.95** | **0.9** | **0.7513** | **0.9987** | **1** | **0.9091** | **0.9545** |
| **GB** | shape | 0.55 | 0.1 | 0.3153 | 0.7694 | 0.5556 | 0.5455 | 0.5505 |
|  | form | 0.85 | 0.7 | 0.6211 | 0.9679 | 0.8889 | 0.8182 | 0.8535 |
| **NB** | shape | 0.55 | 0.1176 | 0.3153 | 0.7694 | 0.6667 | 0.4545 | 0.560 |
|  | form | 0.85 | 0.7 | 0.6211 | 0.9679 | 0.8889 | 0.8182 | 0.8535 |
| **LDA** | shape | 0.75 | 0.5098 | 0.509 | 0.9134 | 0.8889 | 0.6364 | 0.7626 |
|  | form | **0.95** | **0.898** | **0.7513** | **0.9987** | **0.8889** | **1** | **0.944** |
| **PLS** | shape | **1** | **1** | **0.8316** | **1** | **1** | **1** | **1** |
|  | form | **0.9** | **0.802** | **0.683** | **0.9877** | **1** | **0.8182** | **0.9091** |
| **SVMl** | shape | **0.9** | **0.798** | **0.683** | **0.9877** | **0.8889** | **0.9091** | **0.899** |
|  | form | **0.9** | **0.798** | **0.6830** | **0.9877** | **0.8889** | **0.9091** | **0.899** |
| **SVMr** | shape | 0.8 | 0.604 | 0.5634 | 0.9427 | 0.8889 | 0.7273 | 0.8081 |
|  | form | **0.95** | **0.898** | **0.7513** | **0.9987** | **0.8889** | **1** | **0.9444** |
| **NNET** | shape | **0.9** | **0.798** | **0.683** | **0.9877** | **0.8889** | **0.9091** | **0.899** |
|  | form | **0.95** | **0.9** | **0.7513** | **0.9987** | **1** | **0.9091** | **0.9545** |

Table SI 5: Results provided by ML algorithms based on significant PCs in shape and form. Algorithms with accuracy above 90% are highlighted in bold.

| **Model** | **data** | **Accuracy** | **Kappa** | **AccLower** | **AccUpper** | **Sensitivity** | **Specificity** | **BalAccuracy** |
| --- | --- | --- | --- | --- | --- | --- | --- | --- |
| **kNN** | shape | 0.55 | 0.0816 | 0.3153 | 0.7694 | 0.4444 | 0.6364 | 0.5404 |
|  | form | **0.9** | **0.802** | **0.683** | **0.9877** | **1** | **0.8182** | **0.9091** |
| **LGR** | shape | 0.75 | 0.4898 | 0.509 | 0.9134 | 0.6667 | 0.8182 | 0.7424 |
|  | form | **0.95** | **0.898** | **0.7513** | **0.9987** | **0.8889** | **1** | **0.9444** |
| **DTC5.0** | shape | 0.8 | 0.5789 | 0.5634 | 0.9427 | 0.5556 | 1 | 0.7778 |
|  | form | 0.9 | 0.802 | 0.683 | 0.9877 | 1 | 0.8182 | 0.9091 |
| **RF** | shape | **0.9** | **0.798** | **0.683** | **0.9877** | **0.8889** | **0.9091** | **0.899** |
|  | form | **0.95** | **0.9** | **0.7513** | **0.9987** | **1** | **0.9091** | **0.9545** |
| **GB** | shape | 0.65 | 0.3 | 0.4078 | 0.8461 | 0.6667 | 0.6364 | 0.6515 |
|  | form | **0.9** | **0.802** | **0.683** | **0.9877** | **1** | **0.8182** | **0.9091** |
| **NB** | shape | 0.7 | 0.4059 | 0.4572 | 0.8811 | 0.7778 | 0.6364 | 0.7071 |
|  | form | **0.9** | **0.798** | **0.683** | **0.9877** | **0.8889** | **0.9091** | **0.899** |
| **LDA** | shape | 0.8 | 0.5876 | 0.5634 | 0.9427 | 0.6667 | 0.9091 | 0.7879 |
|  | form | **0.9** | **0.802** | **0.683** | **0.9877** | **1** | **0.8182** | **0.9091** |
| **PLS** | shape | 0.8 | 0.5876 | 0.5634 | 0.9427 | 0.6667 | 0.9091 | 0.7879 |
|  | form | **0.9** | **0.802** | **0.683** | **0.9877** | **1** | **0.8182** | **0.9091** |
| **SVMl** | shape | 0.75 | 0.4792 | 0.509 | 0.9134 | 0.5556 | 0.9091 | 0.7323 |
|  | form | **0.95** | **0.898** | **0.7513** | **0.9987** | **0.8889** | **1** | **0.9444** |
| **SVMr** | shape | 0.5 | 0.0989 | 0.272 | 0.728 | 0.00001 | 0.9091 | 0.4545 |
|  | form | 0.85 | 0.7059 | 0.6211 | 0.9679 | 1 | 0.7273 | 0.8636 |
| **NNET** | shape | 0.8 | 0.5876 | 0.5634 | 0.9427 | 0.6667 | 0.9091 | 0.7879 |
|  | form | **0.9** | **0.802** | **0.683** | **0.9877** | **1** | **0.8182** | **0.9091** |

Table SI 6: Classification provided by ML algorithms for the archaeological sample using the complete set of PCs that account for 100% of the total variance in shape and form. The number of samples classified consistently by both anthropological methods and ML algorithms is indicated in brackets.

| **Model** | **Data** | **N male using ML methods** | **N female using ML methods** | **N same sex attribution (%)** |
| --- | --- | --- | --- | --- |
| **kNN** | shape | 6 (3) | 12 (5) | 44.44% |
|  | form | 13 (8) | 5 (3) | 61.11% |
| **LGR** | shape | 9 (5) | 9 (4) | 50% |
|  | form | 13 (8) | 5 (3) | 61.11% |
| **DTC5.0** | shape | 8 (4) | 10 (4) | 44.44% |
|  | form | 12 (7) | 6 (3) | 55.56% |
| **RF** | shape | 9 (6) | 9 (5) | 61.11% |
|  | form | 12 (7) | 6 (3) | 55.56% |
| **GB** | shape | 6 (3) | 12 (5) | 44.44% |
|  | form | 10 (5) | 8 (3) | 44.44% |
| **NB** | shape | 4 (4) | 14 (8) | 66.67% |
|  | form | 11 (6) | 7 (3) | 50% |
| **LDA** | shape | 12 (7) | 6 (3) | 55.56% |
|  | form | 13 (8) | 5 (3) | 61.11% |
| **PLS** | shape | 4 (3) | 14 (7) | 55.56% |
|  | form | 13 (8) | 5 (3) | 61.11% |
| **SVMl** | shape | 6 (5) | 12 (7) | 66.67% |
|  | form | 13 (8) | 5 (3) | 61.11% |
| **SVMr** | shape | 4 (3) | 14 (7) | 55.56% |
|  | form | 17 (10) | 1 (1) | 61.11% |
| **NNET** | shape | 6 (5) | 12 (7) | 66.67% |
|  | form | 13 (8) | 5 (3) | 61.11% |

Table SI 7: Classification provided by ML algorithms for the archaeological sample using the set of PCs that account for 95% of the total variance in shape and form. The number of samples classified consistently by both anthropological methods and ML algorithms is indicated in brackets.

| **Model** | **Data** | **N male using ML methods** | **N female using ML methods** | **N same sex attribution (%)** |
| --- | --- | --- | --- | --- |
| **kNN** | shape | 5 (3) | 13 (6) | 50% |
|  | form | 17 (10) | 1 (1) | 61.11% |
| **LGR** | shape | 7 (5) | 11 (6) | 61.11% |
|  | form | 17 (9) | 1 (1) | 55.56% |
| **DTC5.0** | shape | 12 (6) | 6 (2) | 44.44% |
|  | form | 17 (10) | 1 (1) | 61.11% |
| **RF** | shape | 8 (5) | 10 (4) | 50% |
|  | form | 17 (10) | 1 (1) | 61.11% |
| **GB** | shape | 6 (4) | 12 (6) | 55.56% |
|  | form | 17 (9) | 1 (1) | 55.56% |
| **NB** | shape | 6 (4) | 12 (6) | 55.56% |
|  | form | 15 (9) | 3 (2) | 61.11% |
| **LDA** | shape | 7 (6) | 11 (7) | 72.22% |
|  | form | 12 (9) | 6 (5) | 77.78% |
| **PLS** | shape | 8 (7) | 10 (7) | 77.78% |
|  | form | 17 (10) | 1 (1) | 61.11% |
| **SVMl** | shape | 11 (9) | 7 (6) | 83.33% |
|  | form | 12 (9) | 6 (5) | 77.78% |
| **SVMr** | shape | 11 (6) | 7 (4) | 61.11% |
|  | form | 9 (8) | 9 (7) | 83.33% |
| **NNET** | shape | 10 (8) | 8 (6) | 83.33% |
|  | form | 17 (10) | 1 (1) | 61.11% |

Table SI 8: Classification provided by ML algorithms for the archaeological sample using the significant PCs in shape and form. The number of samples classified consistently by both anthropological methods and ML algorithms is indicated in brackets.

| **Model** | **Data** | **N male using ML methods** | **N female using ML methods** | **N same sex attribution (%)** |
| --- | --- | --- | --- | --- |
| **kNN** | shape | 9 (8) | 9 (7) | 83.33% |
|  | form | 17 (10) | 1 (1) | 61.11% |
| **LGR** | shape | 0 (0) | 18 (8) | 44.44% |
|  | form | 17 (10) | 1 (1) | 61.11% |
| **DTC5.0** | shape | 15 (9) | 3 (2) | 61.11% |
|  | form | 17 (10) | 1 (1) | 61.11% |
| **RF** | shape | 5 (3) | 13 (6) | 50% |
|  | form | 17 (10) | 1 (1) | 61.11% |
| **GB** | shape | 2 (2) | 16 (8) | 55.56% |
|  | form | 17 (10) | 1 (1) | 61.11% |
| **NB** | shape | 7 (5) | 11 (6) | 61.11% |
|  | form | 16 (10) | 2 (2) | 66.67% |
| **LDA** | shape | 0 (0) | 18 (8) | 44.44% |
|  | form | 17 (10) | 1 (1) | 61.11% |
| **PLS** | shape | 0 (0) | 18 (8) | 44.44% |
|  | form | 17 (10) | 1 (1) | 61.11% |
| **SVMl** | shape | 4 (3) | 14 (7) | 55.56% |
|  | form | 15 (9) | 3 (2) | 61.11% |
| **SVMr** | shape | 10 (4) | 8 (2) | 33.33% |
|  | form | 8 (8) | 10 (8) | 88.89% |
| **NNET** | shape | 0 (0) | 18 (8) | 44.44% |
|  | form | 17 (10) | 1 (1) | 61.11% |

Table SI 9: Percentages of probability attribution for the archaeological sample after training the ML models with the complete set of PCs in shape space. Classifications that align with results from anthropological methods are highlighted in green, while probabilities above 90% are marked in red.

| **NNET** | **F** | **M** | **SVMl** | **F** | **M** | **SVMr** | **F** | **M** | **kNN** | **F** | **M** | **LGR** | **F** | **M** | **C50** | **F** | **M** | **RF** | **F** | **M** | **GB** | **F** | **M** | **NB** | **F** | **M** | **LDA** | **F** | **M** | **PLS** | **F** | **M** |
| --- | --- | --- | --- | --- | --- | --- | --- | --- | --- | --- | --- | --- | --- | --- | --- | --- | --- | --- | --- | --- | --- | --- | --- | --- | --- | --- | --- | --- | --- | --- | --- | --- |
| F | **0.998** | 0.002 | F | 0.575 | 0.425 | F | 0.582 | 0.418 | F | 0.545 | 0.455 | F | **1** | 0 | F | 0.816 | 0.184 | M | 0.474 | 0.526 | F | **1** | 0 | F | 0.738 | 0.262 | F | **1** | 0 | F | 0.796 | 0.204 |
| F | **1** | 0 | F | 0.71 | 0.29 | F | 0.678 | 0.322 | F | 0.636 | 0.364 | F | **1** | 0 | F | **0.939** | 0.061 | F | 0.506 | 0.494 | F | **1** | 0 | F | **0.978** | 0.022 | M | 0 | **1** | F | **0.932** | 0.068 |
| F | **1** | 0 | F | 0.608 | 0.392 | F | 0.651 | 0.349 | M | 0.455 | 0.545 | M | 0 | **1** | M | 0.058 | **0.942** | M | 0.442 | 0.558 | F | **1** | 0 | F | **0.985** | 0.015 | M | 0 | **1** | F | 0.875 | 0.125 |
| F | **0.999** | 0.001 | F | **0.908** | 0.092 | F | **0.91** | 0.09 | F | 0.545 | 0.455 | F | **1** | 0 | F | **0.95** | 0.05 | F | 0.606 | 0.394 | F | **1** | 0 | F | **0.963** | 0.037 | M | 0.005 | **0.995** | F | 0.865 | 0.135 |
| M | 0 | **1** | M | 0.049 | **0.951** | M | 0.107 | 0.893 | F | 0.545 | 0.455 | F | **1** | 0 | M | 0.058 | **0.942** | M | 0.262 | 0.738 | M | 0.177 | 0.823 | M | 0.085 | **0.915** | M | 0 | **1** | M | 0.404 | 0.596 |
| M | 0.38 | 0.62 | M | 0.447 | 0.553 | F | 0.652 | 0.348 | M | 0.364 | 0.636 | M | 0 | **1** | M | 0.058 | **0.942** | M | 0.416 | 0.584 | F | **0.999** | 0.001 | F | 0.843 | 0.157 | F | **1** | 0 | F | 0.526 | 0.474 |
| F | **1** | 0 | F | **0.909** | 0.091 | F | 0.889 | 0.111 | M | 0.455 | 0.545 | F | **1** | 0 | M | 0.058 | **0.942** | M | 0.448 | 0.552 | F | **1** | 0 | F | 0.569 | 0.431 | F | **0.999** | 0.001 | F | **0.905** | 0.095 |
| F | **0.978** | 0.022 | F | 0.715 | 0.285 | F | 0.782 | 0.218 | F | 0.545 | 0.455 | F | **1** | 0 | F | **0.939** | 0.061 | F | 0.648 | 0.352 | F | **1** | 0 | F | **0.994** | 0.006 | F | **1** | 0 | F | 0.701 | 0.299 |
| F | **0.999** | 0.001 | F | 0.773 | 0.227 | F | 0.81 | 0.19 | F | 0.545 | 0.455 | M | 0 | **1** | F | **0.95** | 0.05 | F | 0.528 | 0.472 | M | 0.023 | **0.977** | F | **0.996** | 0.004 | F | **1** | 0 | F | 0.743 | 0.257 |
| M | 0.01 | **0.99** | F | 0.830 | 0.170 | F | 0.787 | 0.213 | F | 0.545 | 0.455 | M | 0 | **1** | F | **0.95** | 0.05 | F | 0.504 | 0.496 | F | **0.991** | 0.009 | F | **0.986** | 0.014 | M | 0.365 | 0.635 | M | 0.399 | 0.601 |
| F | **1** | 0 | M | 0.271 | 0.729 | M | 0.473 | 0.527 | M | 0.455 | 0.545 | F | **1** | 0 | F | **0.939** | 0.061 | F | 0.520 | 0.480 | M | 0.015 | **0.985** | F | 0.842 | 0.158 | F | **1** | 0 | F | 0.839 | 0.161 |
| M | 0 | **1** | M | 0.31 | 0.69 | M | 0.337 | 0.663 | M | 0.455 | 0.545 | M | 0 | **1** | M | 0.058 | **0.942** | M | 0.178 | 0.822 | M | 0 | **1** | M | 0.217 | 0.783 | M | 0 | **1** | M | 0.281 | 0.719 |
| F | **1** | 0 | F | 0.855 | 0.145 | F | 0.851 | 0.149 | F | 0.545 | 0.455 | F | **1** | 0 | M | 0.058 | **0.942** | F | 0.508 | 0.492 | F | **1** | 0 | F | **1** | 0 | M | 0 | **1** | F | 0.855 | 0.145 |
| M | 0.325 | 0.675 | M | 0.497 | 0.503 | F | 0.571 | 0.429 | F | 0.545 | 0.455 | F | **1** | 0 | F | 0.816 | 0.184 | F | 0.508 | 0.492 | F | **0.998** | 0.002 | M | 0.141 | 0.859 | M | 0 | **1** | F | 0.601 | 0.399 |
| M | 0.264 | 0.736 | M | 0.298 | 0.702 | M | 0.265 | 0.735 | M | 0.455 | 0.545 | M | 0 | **1** | F | **0.95** | 0.05 | F | 0.564 | 0.436 | F | **1** | 0 | M | 0.017 | **0.983** | M | 0 | **1** | M | 0.446 | 0.554 |
| F | **0.971** | 0.029 | F | 0.82 | 0.18 | F | 0.785 | 0.215 | F | 0.545 | 0.455 | M | 0 | **1** | M | 0.058 | **0.942** | M | 0.298 | 0.702 | F | **0.999** | 0.001 | F | 0.808 | 0.192 | M | 0 | **1** | F | 0.621 | 0.379 |
| F | **0.997** | 0.003 | F | 0.545 | 0.455 | F | 0.521 | 0.479 | F | 0.545 | 0.455 | M | 0 | **1** | M | 0.058 | **0.942** | M | 0.402 | 0.598 | M | 0.319 | 0.681 | F | 0.776 | 0.224 | M | 0 | **1** | F | 0.711 | 0.289 |
| F | **1** | 0 | F | 0.57 | 0.43 | F | 0.549 | 0.451 | F | 0.636 | 0.364 | M | 0 | **1** | F | **0.95** | 0.05 | M | 0.482 | 0.518 | M | 0 | **1** | F | **0.988** | 0.012 | M | 0 | **1** | F | 0.729 | 0.271 |

Table SI 10: Percentages of probability attribution for the archaeological sample after training the ML models with the set of PCs accounting for 95% of the total variance in shape space. Classifications that align with results from anthropological methods are highlighted in green, while probabilities above 90% are marked in red.

| **NNET** | **F** | **M** | **SVMl** | **F** | **M** | **SVMr** | **F** | **M** | **kNN** | **F** | **M** | **LGR** | **F** | **M** | **C50** | **F** | **M** | **RF** | **F** | **M** | **GB** | **F** | **M** | **NB** | **F** | **M** | **LDA** | **F** | **M** | **PLS** | **F** | **M** |
| --- | --- | --- | --- | --- | --- | --- | --- | --- | --- | --- | --- | --- | --- | --- | --- | --- | --- | --- | --- | --- | --- | --- | --- | --- | --- | --- | --- | --- | --- | --- | --- | --- |
| M | 0.441 | 0.559 | M | 0.420 | 0.580 | M | 0.486 | 0.514 | M | 0.474 | 0.526 | F | **1** | 0 | F | **0.908** | 0.092 | F | 0.594 | 0.406 | F | 0.896 | 0.104 | F | 0.684 | 0.316 | M | 0.195 | 0.805 | M | 0.499 | 0.501 |
| F | **0.914** | 0.086 | F | 0.722 | 0.278 | F | 0.720 | 0.280 | F | 0.579 | 0.421 | F | **1** | 0 | F | **0.939** | 0.061 | F | 0.626 | 0.374 | F | **0.997** | 0.003 | F | **0.982** | 0.018 | F | **1** | 0 | F | 0.794 | 0.206 |
| F | 0.849 | 0.151 | F | 0.606 | 0.394 | M | 0.473 | 0.527 | F | 0.579 | 0.421 | F | **1** | 0 | M | 0.132 | 0.868 | F | 0.618 | 0.382 | F | **1** | 0 | F | 0.616 | 0.384 | F | **1** | 0 | F | 0.737 | 0.263 |
| M | 0.405 | 0.595 | M | 0.452 | 0.548 | F | 0.525 | 0.475 | F | 0.579 | 0.421 | F | **1** | 0 | M | 0.132 | 0.868 | F | 0.608 | 0.392 | F | **1** | 0 | F | 0.631 | 0.369 | F | 0.784 | 0.216 | M | 0.471 | 0.529 |
| M | 0.039 | **0.961** | M | 0.081 | **0.919** | M | 0.295 | 0.705 | F | 0.526 | 0.474 | M | 0 | **1** | M | 0.132 | 0.868 | M | 0.282 | 0.718 | M | 0.060 | **0.940** | M | 0.044 | **0.956** | M | 0 | **1** | M | 0.305 | 0.695 |
| M | 0.156 | 0.844 | M | 0.331 | 0.669 | M | 0.343 | 0.657 | M | 0.474 | 0.526 | M | 0 | **1** | M | 0.132 | 0.868 | M | 0.404 | 0.596 | F | 0.849 | 0.151 | F | 0.634 | 0.366 | M | 0.152 | 0.848 | M | 0.393 | 0.607 |
| F | 0.859 | 0.141 | F | 0.582 | 0.418 | F | 0.607 | 0.393 | F | 0.526 | 0.474 | F | **1** | 0 | M | 0.132 | 0.868 | F | 0.622 | 0.378 | F | **1** | 0 | F | **0.910** | 0.090 | F | **1** | 0 | F | 0.762 | 0.238 |
| M | 0.452 | 0.548 | M | 0.200 | 0.800 | M | 0.371 | 0.629 | F | 0.579 | 0.421 | M | 0 | **1** | F | **0.939** | 0.061 | F | 0.648 | 0.352 | F | **0.996** | 0.004 | F | **0.975** | 0.025 | M | 0.273 | 0.727 | F | 0.625 | 0.375 |
| F | 0.749 | 0.251 | F | 0.520 | 0.480 | M | 0.371 | 0.629 | M | 0.474 | 0.526 | F | **1** | 0 | M | 0.025 | **0.975** | M | 0.406 | 0.594 | M | 0.006 | **0.994** | M | 0.001 | **0.999** | F | **0.991** | 0.009 | F | 0.726 | 0.274 |
| M | 0.086 | **0.914** | M | 0.151 | 0.849 | M | 0.251 | 0.749 | M | 0.474 | 0.526 | M | 0 | **1** | M | 0.025 | **0.975** | M | 0.204 | 0.796 | M | 0.001 | **0.999** | M | 0.017 | **0.983** | M | 0 | **1** | M | 0.354 | 0.646 |
| F | 0.824 | 0.176 | F | 0.548 | 0.452 | M | 0.412 | 0.588 | F | 0.579 | 0.421 | M | 0 | **1** | F | **0.939** | 0.061 | F | 0.606 | 0.394 | F | 0.517 | 0.483 | F | **0.989** | 0.011 | F | **0.981** | 0.019 | F | 0.751 | 0.249 |
| M | 0.023 | **0.977** | M | 0.081 | **0.919** | M | 0.223 | 0.777 | M | 0.421 | 0.579 | M | 0 | **1** | M | 0.025 | **0.975** | M | 0.142 | 0.858 | M | 0 | **1** | M | 0 | **1** | M | 0 | **1** | M | 0.272 | 0.728 |
| F | **0.916** | 0.084 | F | 0.746 | 0.254 | F | 0.586 | 0.414 | F | 0.579 | 0.421 | F | **1** | 0 | M | 0.132 | 0.868 | F | 0.670 | 0.330 | F | **0.996** | 0.004 | F | 0.816 | 0.184 | F | **1** | 0 | F | 0.795 | 0.205 |
| M | 0.211 | 0.789 | M | 0.229 | 0.771 | M | 0.422 | 0.578 | F | 0.526 | 0.474 | M | 0 | **1** | F | **0.908** | 0.092 | F | 0.568 | 0.432 | M | 0.068 | **0.932** | F | **0.969** | 0.031 | M | 0.001 | **0.999** | M | 0.465 | 0.535 |
| M | 0.316 | 0.684 | M | 0.447 | 0.553 | M | 0.432 | 0.568 | F | 0.526 | 0.474 | F | **1** | 0 | F | **0.931** | 0.069 | F | 0.580 | 0.420 | F | **0.998** | 0.002 | M | 0.122 | 0.878 | F | 0.728 | 0.272 | M | 0.423 | 0.577 |
| Fe | 0.602 | 0.398 | M | 0.389 | 0.611 | F | 0.501 | 0.499 | F | 0.632 | 0.368 | F | **1** | 0 | M | 0.025 | **0.975** | M | 0.376 | 0.624 | F | 0.789 | 0.211 | M | 0.094 | **0.906** | F | **0.964** | 0.036 | F | 0.648 | 0.352 |
| F | 0.613 | 0.387 | M | 0.495 | 0.505 | F | 0.581 | 0.419 | F | 0.526 | 0.474 | F | **1** | 0 | M | 0.025 | **0.975** | M | 0.440 | 0.560 | F | **0.973** | 0.027 | F | 0.752 | 0.248 | F | **1** | 0 | F | 0.532 | 0.468 |
| F | **0.956** | 0.044 | F | 0.756 | 0.244 | F | 0.715 | 0.285 | F | 0.579 | 0.421 | F | **1** | 0 | M | 0.025 | **0.975** | M | 0.444 | 0.556 | M | 0.001 | **0.999** | F | 0.501 | 0.499 | F | **1** | 0 | F | 0.797 | 0.203 |

Table SI 11: Percentages of probability attribution for the archaeological sample after training the ML models with the set of PCs accounting for 90% of the total variance in shape space. Classifications that align with results from anthropological methods are highlighted in green, while probabilities above 90% are marked in red.

| **NNET** | **F** | **M** | **SVMl** | **F** | **M** | **SVMr** | **F** | **M** | **kNN** | **F** | **M** | **LGR** | **F** | **M** | **C50** | **F** | **M** | **RF** | **F** | **M** | **GB** | **F** | **M** | **NB** | **F** | **M** | **LDA** | **F** | **M** | **PLS** | **F** | **M** |
| --- | --- | --- | --- | --- | --- | --- | --- | --- | --- | --- | --- | --- | --- | --- | --- | --- | --- | --- | --- | --- | --- | --- | --- | --- | --- | --- | --- | --- | --- | --- | --- | --- |
| M | 0.374 | 0.626 | M | 0.066 | **0.934** | F | 0.503 | 0.497 | F | 0.545 | 0.455 | M | 0 | **1** | F | **0.908** | 0.092 | M | 0.468 | 0.532 | M | 0.086 | **0.914** | F | 0.586 | 0.414 | M | 0.024 | **0.976** | M | 0.455 | 0.545 |
| F | 0.822 | 0.178 | M | 0.491 | 0.509 | F | 0.788 | 0.212 | F | 0.727 | 0.273 | M | 0 | **1** | F | **0.939** | 0.061 | F | 0.692 | 0.308 | F | **0.990** | 0.010 | F | **0.997** | 0.003 | F | **0.969** | 0.031 | F | 0.780 | 0.220 |
| F | 0.830 | 0.170 | M | 0.318 | 0.682 | F | 0.632 | 0.368 | M | 0.455 | 0.545 | M | 0 | **1** | M | 0.066 | **0.934** | F | 0.654 | 0.346 | F | **1** | 0 | F | **0.991** | 0.009 | F | **0.985** | 0.015 | F | 0.739 | 0.261 |
| M | 0.421 | 0.579 | M | 0.181 | 0.819 | F | 0.617 | 0.383 | F | 0.545 | 0.455 | M | 0 | **1** | M | 0.066 | **0.934** | F | 0.506 | 0.494 | F | **0.997** | 0.003 | F | 0.640 | 0.360 | M | 0.296 | 0.704 | M | 0.472 | 0.528 |
| M | 0.112 | 0.888 | M | 0.002 | **0.998** | M | 0.365 | 0.635 | M | 0.455 | 0.545 | M | 0 | **1** | M | 0.066 | **0.934** | M | 0.250 | 0.750 | M | 0.073 | **0.927** | F | 0.610 | 0.390 | M | 0 | **1** | M | 0.281 | 0.719 |
| M | 0.249 | 0.751 | M | 0.028 | **0.972** | M | 0.312 | 0.688 | M | 0.364 | 0.636 | M | 0 | **1** | M | 0.066 | **0.934** | M | 0.292 | 0.708 | M | 0.027 | **0.973** | M | 0.425 | 0.575 | M | 0.010 | **0.990** | M | 0.390 | 0.610 |
| F | 0.767 | 0.233 | M | 0.217 | 0.783 | F | 0.630 | 0.370 | M | 0.455 | 0.545 | M | 0 | **1** | M | 0.066 | **0.934** | F | 0.500 | 0.500 | F | **0.992** | 0.008 | F | **0.964** | 0.036 | F | 0.827 | 0.173 | F | 0.738 | 0.262 |
| F | 0.588 | 0.412 | M | 0.046 | **0.954** | M | 0.483 | 0.517 | F | 0.545 | 0.455 | M | 0 | **1** | F | **0.939** | 0.061 | F | 0.536 | 0.464 | F | **0.991** | 0.009 | F | **0.992** | 0.008 | M | 0.061 | **0.939** | F | 0.616 | 0.384 |
| F | 0.772 | 0.228 | F | 0.689 | 0.311 | F | 0.592 | 0.408 | F | 0.545 | 0.455 | F | **1** | 0 | F | 0.827 | 0.173 | M | 0.490 | 0.510 | F | 0.890 | 0.110 | F | 0.879 | 0.121 | F | **0.989** | 0.011 | F | 0.725 | 0.275 |
| M | 0.304 | 0.696 | M | 0.032 | **0.968** | M | 0.376 | 0.624 | F | 0.545 | 0.455 | M | 0 | **1** | F | 0.827 | 0.173 | M | 0.370 | 0.630 | M | 0.046 | **0.954** | F | 0.512 | 0.488 | M | 0.026 | **0.974** | M | 0.407 | 0.593 |
| F | 0.755 | 0.245 | M | 0.430 | 0.570 | M | 0.494 | 0.506 | F | 0.545 | 0.455 | M | 0 | **1** | F | **0.939** | 0.061 | F | 0.540 | 0.460 | M | 0.039 | **0.961** | F | **0.982** | 0.018 | F | 0.793 | 0.207 | F | 0.724 | 0.276 |
| M | 0.132 | 0.868 | M | 0.011 | **0.989** | M | 0.258 | 0.742 | M | 0.455 | 0.545 | M | 0 | **1** | M | 0.074 | **0.926** | M | 0.260 | 0.740 | M | 0 | **1** | M | 0.342 | 0.658 | M | 0 | **1** | M | 0.314 | 0.686 |
| F | 0.815 | 0.185 | F | 0.526 | 0.474 | F | 0.705 | 0.295 | F | 0.545 | 0.455 | F | **1** | 0 | M | 0.066 | **0.934** | F | 0.570 | 0.430 | F | **0.988** | 0.012 | F | **0.995** | 0.005 | F | **0.993** | 0.007 | F | 0.761 | 0.239 |
| M | 0.359 | 0.641 | M | 0.162 | 0.838 | M | 0.381 | 0.619 | F | 0.636 | 0.364 | F | **1** | 0 | F | **0.908** | 0.092 | F | 0.586 | 0.414 | F | **0.957** | 0.043 | F | 0.782 | 0.218 | M | 0.007 | **0.993** | M | 0.476 | 0.524 |
| M | 0.430 | 0.570 | M | 0.282 | 0.718 | F | 0.534 | 0.466 | M | 0.455 | 0.545 | F | **1** | 0 | F | 0.827 | 0.173 | F | 0.540 | 0.460 | F | **1** | 0 | M | 0.399 | 0.601 | F | 0.661 | 0.339 | M | 0.449 | 0.551 |
| F | 0.704 | 0.296 | M | 0.126 | 0.874 | F | 0.669 | 0.331 | F | 0.545 | 0.455 | M | 0 | **1** | F | 0.827 | 0.173 | F | 0.576 | 0.424 | F | **0.994** | 0.006 | F | **0.979** | 0.021 | F | 0.573 | 0.427 | F | 0.677 | 0.323 |
| M | 0.499 | 0.501 | M | 0.163 | 0.837 | M | 0.480 | 0.520 | F | 0.545 | 0.455 | M | 0 | **1** | M | 0.066 | **0.934** | F | 0.512 | 0.488 | F | **0.992** | 0.008 | F | 0.853 | 0.147 | M | 0.365 | 0.635 | F | 0.509 | 0.491 |
| F | 0.851 | 0.149 | F | 0.872 | 0.128 | F | 0.765 | 0.235 | F | 0.636 | 0.364 | F | **1** | 0 | F | 0.827 | 0.173 | F | 0.558 | 0.442 | F | 0.737 | 0.263 | F | **0.972** | 0.028 | F | **0.993** | 0.007 | F | 0.764 | 0.236 |

Table SI 12: Percentages of probability attribution for the archaeological sample after training the ML models with the significant PCs in shape space. Classifications that align with results from anthropological methods are highlighted in green, while probabilities above 90% are marked in red.

| **NNET** | **F** | **M** | **SVMl** | **F** | **M** | **SVMr** | **F** | **M** | **kNN** | **F** | **M** | **LGR** | **F** | **M** | **C50** | **F** | **M** | **RF** | **F** | **M** | **GB** | **F** | **M** | **NB** | **F** | **M** | **LDA** | **F** | **M** | **PLS** | **F** | **M** |
| --- | --- | --- | --- | --- | --- | --- | --- | --- | --- | --- | --- | --- | --- | --- | --- | --- | --- | --- | --- | --- | --- | --- | --- | --- | --- | --- | --- | --- | --- | --- | --- | --- |
| F | 0.598 | 0.402 | F | 0.525 | 0.475 | F | 0.505 | 0.495 | M | 0.2 | 0.8 | F | 0.665 | 0.335 | M | 0.336 | 0.664 | F | 0.556 | 0.444 | F | 0.849 | 0.151 | M | 0.470 | 0.530 | F | 0.665 | 0.335 | F | 0.567 | 0.433 |
| F | 0.878 | 0.122 | F | 0.759 | 0.241 | M | 0.440 | 0.560 | F | 0.6 | 0.4 | F | **0.931** | 0.069 | F | **0.939** | 0.061 | F | 0.786 | 0.214 | F | **0.949** | 0.051 | F | **0.994** | 0.006 | F | **0.926** | 0.074 | F | 0.728 | 0.272 |
| F | **0.927** | 0.073 | F | 0.837 | 0.163 | M | 0.486 | 0.514 | F | 0.6 | 0.4 | F | **0.968** | 0.032 | M | 0.336 | 0.664 | F | 0.752 | 0.248 | F | **0.997** | 0.003 | F | 0.694 | 0.306 | F | **0.964** | 0.036 | F | 0.783 | 0.217 |
| F | 0.532 | 0.468 | M | 0.492 | 0.508 | F | 0.532 | 0.468 | M | 0.4 | 0.6 | F | 0.580 | 0.420 | M | 0.336 | 0.664 | F | 0.538 | 0.462 | F | 0.822 | 0.178 | M | 0.167 | 0.833 | F | 0.579 | 0.421 | F | 0.532 | 0.468 |
| F | 0.730 | 0.270 | F | 0.614 | 0.386 | F | 0.505 | 0.495 | M | 0.2 | 0.8 | F | 0.820 | 0.180 | M | 0.336 | 0.664 | M | 0.416 | 0.584 | M | 0.467 | 0.533 | M | 0.264 | 0.736 | F | 0.801 | 0.199 | F | 0.632 | 0.368 |
| F | 0.636 | 0.364 | F | 0.557 | 0.443 | F | 0.554 | 0.446 | M | 0.4 | 0.6 | F | 0.710 | 0.290 | M | 0.336 | 0.664 | F | 0.702 | 0.298 | F | **0.951** | 0.049 | F | 0.737 | 0.263 | F | 0.693 | 0.307 | F | 0.579 | 0.421 |
| F | **0.904** | 0.096 | F | 0.805 | 0.195 | M | 0.494 | 0.506 | F | 0.6 | 0.4 | F | **0.951** | 0.049 | M | 0.336 | 0.664 | F | 0.754 | 0.246 | F | **0.971** | 0.029 | F | 0.663 | 0.337 | F | **0.946** | 0.054 | F | 0.753 | 0.247 |
| F | **0.937** | 0.063 | F | 0.850 | 0.150 | M | 0.478 | 0.522 | F | 0.6 | 0.4 | F | **0.974** | 0.026 | F | **0.939** | 0.061 | F | 0.814 | 0.186 | F | **0.977** | 0.023 | F | 0.811 | 0.189 | F | **0.971** | 0.029 | F | 0.797 | 0.203 |
| F | 0.818 | 0.182 | F | 0.675 | 0.325 | M | 0.464 | 0.536 | F | 0.6 | 0.4 | F | 0.890 | 0.110 | M | 0.336 | 0.664 | M | 0.484 | 0.516 | F | 0.603 | 0.397 | M | 0.193 | 0.807 | F | 0.871 | 0.129 | F | 0.678 | 0.322 |
| F | 0.693 | 0.307 | F | 0.611 | 0.389 | F | 0.535 | 0.465 | F | 0.6 | 0.4 | F | 0.763 | 0.237 | M | 0.336 | 0.664 | F | 0.706 | 0.294 | F | 0.885 | 0.115 | F | 0.747 | 0.253 | F | 0.758 | 0.242 | F | 0.609 | 0.391 |
| F | 0.859 | 0.141 | F | 0.749 | 0.251 | M | 0.432 | 0.568 | F | 0.6 | 0.4 | F | **0.912** | 0.088 | F | **0.939** | 0.061 | F | 0.770 | 0.230 | F | 0.643 | 0.357 | F | **0.969** | 0.031 | F | **0.910** | 0.090 | F | 0.711 | 0.289 |
| F | 0.529 | 0.471 | M | 0.438 | 0.562 | F | 0.520 | 0.480 | M | 0.4 | 0.6 | F | 0.609 | 0.391 | M | 0.336 | 0.664 | M | 0.366 | 0.634 | M | 0.341 | 0.659 | M | 0.296 | 0.704 | F | 0.566 | 0.434 | F | 0.527 | 0.473 |
| F | 0.802 | 0.198 | F | 0.674 | 0.326 | F | 0.507 | 0.493 | M | 0.4 | 0.6 | F | 0.882 | 0.118 | M | 0.336 | 0.664 | M | 0.346 | 0.654 | F | 0.772 | 0.228 | M | 0.266 | 0.734 | F | 0.864 | 0.136 | F | 0.673 | 0.327 |
| F | 0.678 | 0.322 | F | 0.571 | 0.429 | M | 0.497 | 0.503 | M | 0.4 | 0.6 | F | 0.752 | 0.248 | M | 0.336 | 0.664 | F | 0.566 | 0.434 | F | 0.724 | 0.276 | F | 0.645 | 0.355 | F | 0.742 | 0.258 | F | 0.602 | 0.398 |
| F | 0.521 | 0.479 | M | 0.478 | 0.522 | F | 0.573 | 0.427 | M | 0.4 | 0.6 | F | 0.578 | 0.422 | M | 0.336 | 0.664 | F | 0.590 | 0.410 | F | 0.867 | 0.133 | F | 0.650 | 0.350 | F | 0.565 | 0.435 | F | 0.526 | 0.474 |
| F | 0.879 | 0.121 | F | 0.778 | 0.222 | M | 0.493 | 0.507 | M | 0.4 | 0.6 | F | **0.935** | 0.065 | M | 0.336 | 0.664 | F | 0.702 | 0.298 | F | **0.989** | 0.011 | F | 0.744 | 0.256 | F | **0.930** | 0.070 | F | 0.733 | 0.267 |
| F | 0.568 | 0.432 | M | 0.488 | 0.512 | M | 0.485 | 0.515 | F | 0.6 | 0.4 | F | 0.629 | 0.371 | M | 0.336 | 0.664 | F | 0.580 | 0.420 | F | 0.741 | 0.259 | F | 0.698 | 0.302 | F | 0.621 | 0.379 | F | 0.549 | 0.451 |
| F | 0.756 | 0.244 | F | 0.633 | 0.367 | M | 0.484 | 0.516 | F | 0.6 | 0.4 | F | 0.834 | 0.166 | M | 0.336 | 0.664 | M | 0.488 | 0.512 | F | 0.645 | 0.355 | M | 0.042 | **0.958** | F | 0.810 | 0.190 | F | 0.638 | 0.362 |

Table SI 13: Percentages of probability attribution for the archaeological sample after training the ML models with the complete set of PCs in form space. Classifications that align with results from anthropological methods are highlighted in green, while probabilities above 90% are marked in red.

| **kNN** | **F** | **M** | **LGR** | **F** | **M** | **C50** | **F** | **M** | **RF** | **F** | **M** | **GB** | **F** | **M** | **NB** | **F** | **M** | **LDA** | **F** | **M** | **PLS** | **F** | **M** | **SVMl** | **F** | **M** | **SVMr** | **F** | **M** | **NNET** | **F** | **M** |
| --- | --- | --- | --- | --- | --- | --- | --- | --- | --- | --- | --- | --- | --- | --- | --- | --- | --- | --- | --- | --- | --- | --- | --- | --- | --- | --- | --- | --- | --- | --- | --- | --- |
| M | 0.4 | 0.6 | F | 0.770 | 0.230 | F | **0.939** | 0.061 | F | 0.866 | 0.134 | F | **0.993** | 0.007 | F | **0.999** | 0.001 | F | 0.796 | 0.204 | F | 0.629 | 0.371 | F | 0.657 | 0.343 | M | 0.464 | 0.536 | F | 0.754 | 0.246 |
| F | 0.6 | 0.4 | F | 0.870 | 0.130 | F | **0.939** | 0.061 | F | 0.590 | 0.410 | F | 0.703 | 0.297 | F | **0.971** | 0.029 | F | 0.869 | 0.131 | F | 0.676 | 0.324 | F | 0.670 | 0.330 | F | 0.511 | 0.489 | F | 0.827 | 0.173 |
| F | 0.6 | 0.4 | M | 0.324 | 0.676 | M | 0.336 | 0.664 | M | 0.260 | 0.740 | M | 0.223 | 0.777 | M | 0.163 | 0.837 | M | 0.331 | 0.669 | M | 0.433 | 0.567 | M | 0.426 | 0.574 | M | 0.417 | 0.583 | M | 0.351 | 0.649 |
| M | 0.4 | 0.6 | M | 0.318 | 0.682 | F | **0.939** | 0.061 | F | 0.690 | 0.310 | F | **0.984** | 0.016 | F | 0.821 | 0.179 | M | 0.351 | 0.649 | M | 0.441 | 0.559 | M | 0.459 | 0.541 | M | 0.444 | 0.556 | M | 0.378 | 0.622 |
| M | 0.2 | 0.8 | M | 0.244 | 0.756 | M | 0.336 | 0.664 | M | 0.336 | 0.664 | M | 0.465 | 0.535 | M | 0.250 | 0.750 | M | 0.262 | 0.738 | M | 0.402 | 0.598 | M | 0.414 | 0.586 | M | 0.444 | 0.556 | M | 0.305 | 0.695 |
| M | 0 | **1** | M | 0.050 | **0.950** | M | 0.336 | 0.664 | M | 0.396 | 0.604 | M | 0.397 | 0.603 | F | **0.990** | 0.010 | M | 0.056 | **0.944** | M | 0.252 | 0.748 | M | 0.234 | 0.766 | M | 0.390 | 0.610 | M | 0.093 | **0.907** |
| M | 0.2 | 0.8 | M | 0.130 | 0.870 | M | 0.336 | 0.664 | M | 0.178 | 0.822 | M | 0.036 | **0.964** | M | 0.070 | **0.930** | M | 0.138 | 0.862 | M | 0.330 | 0.670 | M | 0.307 | 0.693 | M | 0.408 | 0.592 | M | 0.178 | 0.822 |
| F | 0.6 | 0.4 | F | 0.579 | 0.421 | M | 0.336 | 0.664 | M | 0.324 | 0.676 | M | 0.390 | 0.610 | M | 0.202 | 0.798 | F | 0.585 | 0.415 | F | 0.534 | 0.466 | F | 0.531 | 0.469 | M | 0.398 | 0.602 | F | 0.560 | 0.440 |
| M | 0.4 | 0.6 | M | 0.217 | 0.783 | M | 0.336 | 0.664 | M | 0.142 | 0.858 | M | 0.028 | **0.972** | M | 0.116 | 0.884 | M | 0.210 | 0.790 | M | 0.375 | 0.625 | M | 0.330 | 0.670 | M | 0.390 | 0.610 | M | 0.251 | 0.749 |
| M | 0.2 | 0.8 | M | 0.224 | 0.776 | M | 0.336 | 0.664 | M | 0.354 | 0.646 | M | 0.394 | 0.606 | M | 0.369 | 0.631 | M | 0.247 | 0.753 | M | 0.394 | 0.606 | M | 0.381 | 0.619 | M | 0.408 | 0.592 | M | 0.273 | 0.727 |
| M | 0.2 | 0.8 | F | **0.913** | 0.087 | F | **0.939** | 0.061 | F | 0.702 | 0.298 | F | 0.591 | 0.409 | F | **0.990** | 0.010 | F | **0.913** | 0.087 | F | 0.714 | 0.286 | F | 0.677 | 0.323 | M | 0.409 | 0.591 | F | 0.860 | 0.140 |
| M | 0.2 | 0.8 | M | 0.057 | **0.943** | M | 0.336 | 0.664 | M | 0.248 | 0.752 | M | 0.386 | 0.614 | M | 0.191 | 0.809 | M | 0.062 | **0.938** | M | 0.258 | 0.742 | M | 0.245 | 0.755 | M | 0.435 | 0.565 | M | 0.104 | 0.896 |
| M | 0.4 | 0.6 | M | 0.299 | 0.701 | M | 0.336 | 0.664 | M | 0.202 | 0.798 | M | 0.074 | **0.926** | M | 0.056 | **0.944** | M | 0.303 | 0.697 | M | 0.421 | 0.579 | M | 0.386 | 0.614 | M | 0.416 | 0.584 | M | 0.325 | 0.675 |
| M | 0.4 | 0.6 | M | 0.274 | 0.726 | F | **0.939** | 0.061 | F | 0.776 | 0.224 | F | **0.932** | 0.068 | F | **0.948** | 0.052 | M | 0.301 | 0.699 | M | 0.420 | 0.580 | M | 0.443 | 0.557 | M | 0.456 | 0.544 | M | 0.346 | 0.654 |
| M | 0.4 | 0.6 | M | 0.056 | **0.944** | M | 0.336 | 0.664 | M | 0.470 | 0.530 | F | 0.622 | 0.378 | M | 0.391 | 0.609 | M | 0.065 | **0.935** | M | 0.263 | 0.737 | M | 0.279 | 0.721 | M | 0.450 | 0.550 | M | 0.109 | 0.891 |
| M | 0 | **1** | M | 0.182 | 0.818 | M | 0.336 | 0.664 | M | 0.454 | 0.546 | F | 0.727 | 0.273 | M | 0.346 | 0.654 | M | 0.203 | 0.797 | M | 0.371 | 0.629 | M | 0.383 | 0.617 | M | 0.442 | 0.558 | M | 0.246 | 0.754 |
| F | 0.6 | 0.4 | F | 0.842 | 0.158 | F | **0.939** | 0.061 | F | 0.702 | 0.298 | F | 0.608 | 0.392 | F | **0.945** | 0.055 | F | 0.846 | 0.154 | F | 0.660 | 0.340 | F | 0.664 | 0.336 | M | 0.482 | 0.518 | F | 0.803 | 0.197 |
| F | 0.6 | 0.4 | M | 0.354 | 0.646 | M | 0.336 | 0.664 | M | 0.354 | 0.646 | M | 0.026 | **0.974** | M | 0.231 | 0.769 | M | 0.332 | 0.668 | M | 0.433 | 0.567 | M | 0.392 | 0.608 | M | 0.441 | 0.559 | M | 0.361 | 0.639 |

Table SI 14: Percentages of probability attribution for the archaeological sample after training the ML models with the set of PCs accounting for 95% of the total variance in form space. Classifications that align with results from anthropological methods are highlighted in green, while probabilities above 90% are marked in red.

| **NNET** | **F** | **M** | **SVMl** | **F** | **M** | **SVMr** | **F** | **M** | **kNN** | **F** | **M** | **LGR** | **F** | **M** | **C50** | **F** | **M** | **RF** | **F** | **M** | **GB** | **F** | **M** | **NB** | **F** | **M** | **LDA** | **F** | **M** | **PLS** | **F** | **M** |
| --- | --- | --- | --- | --- | --- | --- | --- | --- | --- | --- | --- | --- | --- | --- | --- | --- | --- | --- | --- | --- | --- | --- | --- | --- | --- | --- | --- | --- | --- | --- | --- | --- |
| M | 0.14 | 0.86 | M | 0.33 | 0.67 | M | 0.416 | 0.584 | M | 0.095 | **0.905** | M | 0 | **1** | M | 0.022 | **0.978** | M | 0.08 | **0.92** | M | 0.452 | 0.548 | M | 0.001 | **0.999** | M | 0.002 | **0.998** | M | 0.229 | 0.771 |
| M | 0.259 | 0.741 | F | 0.557 | 0.443 | F | 0.875 | 0.125 | M | 0.238 | 0.762 | M | 0 | **1** | M | 0.022 | **0.978** | M | 0.084 | **0.916** | M | 0.259 | 0.741 | M | 0.055 | **0.945** | F | 0.879 | 0.121 | M | 0.337 | 0.663 |
| M | 0.327 | 0.673 | F | 0.51 | 0.49 | F | 0.664 | 0.336 | M | 0.429 | 0.571 | M | 0 | **1** | M | 0.022 | **0.978** | M | 0.062 | **0.938** | M | 0.207 | 0.793 | M | 0.183 | 0.817 | M | 0.383 | 0.617 | M | 0.391 | 0.609 |
| M | 0.1 | **0.9** | M | 0.255 | 0.745 | M | 0.091 | **0.909** | M | 0.095 | **0.905** | M | 0 | **1** | M | 0.022 | **0.978** | M | 0.076 | **0.924** | M | 0.484 | 0.516 | M | 0 | **1** | M | 0 | **1** | M | 0.181 | 0.819 |
| M | 0.113 | 0.887 | M | 0.071 | **0.929** | M | 0.225 | 0.775 | M | 0.048 | **0.952** | M | 0 | **1** | M | 0.022 | **0.978** | M | 0.052 | **0.948** | M | 0.122 | 0.878 | M | 0 | **1** | M | 0 | **1** | M | 0.201 | 0.799 |
| M | 0.165 | 0.835 | M | 0.137 | 0.863 | M | 0.053 | **0.947** | M | 0.143 | 0.857 | M | 0 | **1** | M | 0.022 | **0.978** | M | 0.052 | **0.948** | M | 0.174 | 0.826 | M | 0 | **1** | M | 0 | **1** | M | 0.259 | 0.741 |
| M | 0.242 | 0.758 | M | 0.403 | 0.597 | F | 0.508 | 0.492 | M | 0.286 | 0.714 | M | 0 | **1** | M | 0.022 | **0.978** | M | 0.084 | **0.916** | M | 0.357 | 0.643 | M | 0.023 | **0.977** | M | 0.036 | **0.964** | M | 0.322 | 0.678 |
| M | 0.310 | 0.69 | M | 0.253 | 0.747 | F | 0.593 | 0.407 | M | 0.381 | 0.619 | M | 0 | **1** | M | 0.022 | **0.978** | M | 0.062 | **0.938** | M | 0.087 | **0.913** | M | 0.26 | 0.740 | M | 0 | **1** | M | 0.383 | 0.617 |
| M | 0.333 | 0.667 | F | 0.545 | 0.455 | F | 0.599 | 0.401 | M | 0.381 | 0.619 | M | 0 | **1** | M | 0.022 | **0.978** | M | 0.064 | **0.936** | M | 0.208 | 0.792 | M | 0.04 | **0.96** | F | 0.883 | 0.117 | M | 0.39 | 0.61 |
| M | 0.232 | 0.768 | M | 0.214 | 0.786 | M | 0.127 | 0.873 | M | 0.286 | 0.714 | M | 0 | **1** | M | 0.022 | **0.978** | M | 0.062 | **0.938** | M | 0.235 | 0.765 | M | 0.01 | **0.99** | M | 0 | **1** | M | 0.321 | 0.679 |
| F | 0.651 | 0.349 | F | 0.536 | 0.464 | F | 0.763 | 0.237 | F | 0.667 | 0.333 | M | 0 | **1** | F | 0.740 | 0.260 | F | 0.72 | 0.28 | F | 0.613 | 0.387 | F | **0.946** | 0.054 | F | 0.887 | 0.113 | F | 0.629 | 0.371 |
| M | 0.096 | **0.904** | M | 0.15 | 0.85 | M | 0.035 | **0.965** | M | 0.095 | **0.905** | M | 0 | **1** | M | 0.022 | **0.978** | M | 0.048 | **0.952** | M | 0.227 | 0.773 | M | 0 | **1** | M | 0 | **1** | M | 0.174 | 0.826 |
| M | 0.428 | 0.572 | F | 0.678 | 0.322 | F | **0.959** | 0.041 | M | 0.476 | 0.524 | F | **1** | 0 | M | 0.022 | **0.978** | M | 0.07 | **0.93** | M | 0.193 | 0.807 | F | 0.791 | 0.209 | F | **0.998** | 0.002 | M | 0.467 | 0.533 |
| M | 0.063 | **0.937** | M | 0.179 | 0.821 | M | 0.084 | **0.916** | M | 0.095 | **0.905** | M | 0 | **1** | M | 0.022 | **0.978** | M | 0.05 | **0.95** | M | 0.196 | 0.804 | M | 0 | **1** | M | 0 | **1** | M | 0.126 | 0.874 |
| M | 0.063 | **0.937** | M | 0.306 | 0.694 | M | 0.059 | **0.941** | M | 0.095 | **0.905** | M | 0 | **1** | M | 0.022 | **0.978** | M | 0.088 | **0.912** | F | 0.628 | 0.372 | M | 0 | **1** | M | 0 | **1** | M | 0.124 | 0.876 |
| M | 0.123 | 0.877 | M | 0.352 | 0.648 | M | 0.302 | 0.698 | M | 0.143 | 0.857 | M | 0 | **1** | M | 0.022 | **0.978** | M | 0.082 | **0.918** | M | 0.338 | 0.662 | M | 0 | **1** | M | 0.002 | **0.998** | M | 0.209 | 0.791 |
| M | 0.264 | 0.736 | M | 0.463 | 0.537 | F | 0.652 | 0.348 | M | 0.333 | 0.667 | M | 0 | **1** | M | 0.022 | **0.978** | M | 0.074 | **0.926** | M | 0.097 | **0.903** | M | 0.054 | **0.946** | F | 0.523 | 0.477 | M | 0.34 | 0.66 |
| M | 0.382 | 0.618 | F | 0.793 | 0.207 | F | **0.944** | 0.056 | M | 0.476 | 0.524 | F | **1** | 0 | M | 0.022 | **0.978** | M | 0.084 | **0.916** | M | 0.38 | 0.62 | F | 0.736 | 0.264 | F | **1** | 0 | M | 0.421 | 0.579 |

Table SI 15: Percentages of probability attribution for the archaeological sample after training the ML models with the set of PCs accounting for 90% of the total variance in form space. Classifications that align with results from anthropological methods are highlighted in green, while probabilities above 90% are marked in red.

| **NNET** | **F** | **M** | **SVMl** | **F** | **M** | **SVMr** | **F** | **M** | **kNN** | **F** | **M** | **LGR** | **F** | **M** | **C50** | **F** | **M** | **RF** | **F** | **M** | **GB** | **F** | **M** | **NB** | **F** | **M** | **LDA** | **F** | **M** | **PLS** | **F** | **M** |
| --- | --- | --- | --- | --- | --- | --- | --- | --- | --- | --- | --- | --- | --- | --- | --- | --- | --- | --- | --- | --- | --- | --- | --- | --- | --- | --- | --- | --- | --- | --- | --- | --- |
| M | 0.077 | **0.923** | M | 0.231 | 0.769 | M | 0.32 | 0.68 | M | 0.095 | **0.905** | M | 0 | **1** | M | 0.022 | **0.978** | M | 0.172 | 0.828 | M | 0.048 | **0.952** | M | 0 | **1** | M | 0.001 | **0.999** | M | 0.228 | 0.772 |
| M | 0.196 | 0.804 | M | 0.326 | 0.674 | F | 0.673 | 0.327 | M | 0.19 | 0.81 | M | 0.008 | **0.992** | M | 0.022 | **0.978** | M | 0.196 | 0.804 | M | 0.13 | 0.87 | M | 0.03 | **0.97** | M | 0.021 | **0.979** | M | 0.336 | 0.664 |
| M | 0.304 | 0.696 | M | 0.493 | 0.507 | F | 0.77 | 0.23 | M | 0.429 | 0.571 | M | 0.246 | 0.754 | M | 0.022 | **0.978** | M | 0.186 | 0.814 | M | 0.358 | 0.642 | M | 0.191 | 0.809 | M | 0.399 | 0.601 | M | 0.393 | 0.607 |
| M | 0.047 | **0.953** | M | 0.185 | 0.815 | M | 0.218 | 0.782 | M | 0.095 | **0.905** | M | 0 | **1** | M | 0.022 | **0.978** | M | 0.168 | 0.832 | M | 0.11 | 0.89 | M | 0 | **1** | M | 0 | **1** | M | 0.181 | 0.819 |
| M | 0.06 | **0.94** | M | 0.224 | 0.776 | M | 0.351 | 0.649 | M | 0.095 | **0.905** | M | 0 | **1** | M | 0.022 | **0.978** | M | 0.164 | 0.836 | M | 0.059 | **0.941** | M | 0 | **1** | M | 0 | **1** | M | 0.203 | 0.797 |
| M | 0.114 | 0.886 | M | 0.302 | 0.698 | M | 0.328 | 0.672 | M | 0.19 | 0.81 | M | 0.002 | **0.998** | M | 0.022 | **0.978** | M | 0.168 | 0.832 | M | 0.12 | 0.88 | M | 0.001 | **0.999** | M | 0.007 | **0.993** | M | 0.264 | 0.736 |
| M | 0.188 | 0.812 | M | 0.351 | 0.649 | F | 0.543 | 0.457 | M | 0.286 | 0.714 | M | 0.015 | **0.985** | M | 0.022 | **0.978** | M | 0.156 | 0.844 | M | 0.122 | 0.878 | M | 0.017 | **0.983** | M | 0.04 | **0.96** | M | 0.322 | 0.678 |
| M | 0.269 | 0.731 | M | 0.396 | 0.604 | F | 0.695 | 0.305 | M | 0.381 | 0.619 | M | 0.017 | **0.983** | M | 0.022 | **0.978** | M | 0.172 | 0.828 | M | 0.07 | **0.93** | M | 0.1 | **0.9** | M | 0.046 | **0.954** | M | 0.385 | 0.615 |
| M | 0.306 | 0.694 | F | 0.511 | 0.489 | F | 0.641 | 0.359 | M | 0.381 | 0.619 | F | 0.603 | 0.397 | M | 0.022 | **0.978** | M | 0.174 | 0.826 | M | 0.26 | 0.74 | M | 0.186 | 0.814 | F | 0.505 | 0.495 | M | 0.39 | 0.61 |
| M | 0.18 | 0.82 | M | 0.354 | 0.646 | M | 0.498 | 0.502 | M | 0.286 | 0.714 | M | 0.007 | **0.993** | M | 0.022 | **0.978** | M | 0.206 | 0.794 | M | 0.169 | 0.831 | M | 0.024 | **0.976** | M | 0.023 | **0.977** | M | 0.324 | 0.676 |
| F | 0.705 | 0.295 | F | 0.52 | 0.48 | F | 0.727 | 0.273 | F | 0.667 | 0.333 | F | 0.84 | 0.16 | F | 0.740 | 0.260 | F | 0.726 | 0.274 | F | 0.557 | 0.443 | F | **0.95** | 0.05 | F | 0.781 | 0.219 | F | 0.629 | 0.371 |
| M | 0.048 | **0.952** | M | 0.282 | 0.718 | M | 0.263 | 0.737 | M | 0.095 | **0.905** | M | 0 | **1** | M | 0.022 | **0.978** | M | 0.184 | 0.816 | M | 0.14 | 0.86 | M | 0 | **1** | M | 0.001 | **0.999** | M | 0.175 | 0.825 |
| M | 0.409 | 0.591 | M | 0.463 | 0.537 | F | 0.82 | 0.18 | M | 0.476 | 0.524 | M | 0.238 | 0.762 | M | 0.022 | **0.978** | M | 0.178 | 0.822 | M | 0.202 | 0.798 | F | 0.671 | 0.329 | M | 0.32 | 0.68 | M | 0.465 | 0.535 |
| M | 0.023 | **0.977** | M | 0.167 | 0.833 | M | 0.189 | 0.811 | M | 0.095 | **0.905** | M | 0 | **1** | M | 0.022 | **0.978** | M | 0.132 | 0.868 | M | 0.071 | **0.929** | M | 0 | **1** | M | 0 | **1** | M | 0.127 | 0.873 |
| M | 0.024 | **0.976** | M | 0.215 | 0.785 | M | 0.141 | 0.859 | M | 0.136 | 0.864 | M | 0 | **1** | M | 0.022 | **0.978** | M | 0.182 | 0.818 | M | 0.28 | 0.72 | M | 0 | **1** | M | 0 | **1** | M | 0.124 | 0.876 |
| M | 0.066 | **0.934** | M | 0.24 | 0.76 | M | 0.378 | 0.622 | M | 0.095 | **0.905** | M | 0 | **1** | M | 0.022 | **0.978** | M | 0.202 | 0.798 | M | 0.103 | 0.897 | M | 0 | **1** | M | 0.001 | **0.999** | M | 0.21 | 0.79 |
| M | 0.207 | 0.793 | M | 0.372 | 0.628 | F | 0.508 | 0.492 | M | 0.333 | 0.667 | M | 0.057 | **0.943** | M | 0.022 | **0.978** | M | 0.162 | 0.838 | M | 0.086 | **0.914** | M | 0.034 | **0.966** | M | 0.058 | **0.942** | M | 0.34 | 0.66 |
| M | 0.362 | 0.638 | F | 0.551 | 0.449 | F | 0.735 | 0.265 | M | 0.476 | 0.524 | F | 0.855 | 0.145 | M | 0.022 | **0.978** | M | 0.178 | 0.822 | M | 0.312 | 0.688 | F | 0.598 | 0.402 | F | 0.796 | 0.204 | M | 0.419 | 0.581 |

Table SI 16: Percentages of probability attribution for the archaeological sample after training the ML models with the significant PCs in form space. Classifications that align with results from anthropological methods are highlighted in green, while probabilities above 90% are marked in red.

| **kNN** | **F** | **M** | **LGR** | **F** | **M** | **C50** | **F** | **M** | **RF** | **F** | **M** | **GB** | **F** | **M** | **NB** | **F** | **M** | **LDA** | **F** | **M** | **PLS** | **F** | **M** | **SVMl** | **F** | **M** | **SVMr** | **F** | **M** | **NNET** | **F** | **M** |
| --- | --- | --- | --- | --- | --- | --- | --- | --- | --- | --- | --- | --- | --- | --- | --- | --- | --- | --- | --- | --- | --- | --- | --- | --- | --- | --- | --- | --- | --- | --- | --- | --- |
| M | 0 | **1** | M | 0.004 | **0.996** | M | 0.022 | **0.978** | M | 0.174 | 0.826 | M | 0.076 | **0.924** | M | 0 | **1** | M | 0.009 | **0.991** | M | 0.230 | 0.770 | M | 0.129 | 0.871 | M | 0.438 | 0.562 | M | 0.184 | 0.816 |
| M | 0.158 | 0.842 | M | 0.060 | **0.940** | M | 0.022 | **0.978** | M | 0.190 | 0.810 | M | 0.064 | **0.936** | M | 0.033 | **0.967** | M | 0.106 | 0.894 | M | 0.335 | 0.665 | M | 0.308 | 0.692 | F | 0.677 | 0.323 | M | 0.287 | 0.713 |
| M | 0.474 | 0.526 | M | 0.274 | 0.726 | M | 0.022 | **0.978** | M | 0.140 | 0.860 | M | 0.079 | **0.921** | M | 0.111 | 0.889 | M | 0.217 | 0.783 | M | 0.389 | 0.611 | M | 0.446 | 0.554 | F | 0.654 | 0.346 | M | 0.345 | 0.655 |
| M | 0 | **1** | M | 0.001 | **0.999** | M | 0.022 | **0.978** | M | 0.146 | 0.854 | M | 0.074 | **0.926** | M | 0 | **1** | M | 0.003 | **0.997** | M | 0.182 | 0.818 | M | 0.101 | 0.899 | M | 0.241 | 0.759 | M | 0.146 | 0.854 |
| M | 0.053 | **0.947** | M | 0.005 | **0.995** | M | 0.022 | **0.978** | M | 0.156 | 0.844 | M | 0.117 | 0.883 | M | 0 | **1** | M | 0.008 | **0.992** | M | 0.207 | 0.793 | M | 0.169 | 0.831 | M | 0.477 | 0.523 | M | 0.167 | 0.833 |
| M | 0.105 | 0.895 | M | 0.018 | **0.982** | M | 0.022 | **0.978** | M | 0.170 | 0.830 | M | 0.027 | **0.973** | M | 0.001 | **0.999** | M | 0.025 | **0.975** | M | 0.265 | 0.735 | M | 0.214 | 0.786 | M | 0.317 | 0.683 | M | 0.217 | 0.783 |
| M | 0.263 | 0.737 | M | 0.066 | **0.934** | M | 0.022 | **0.978** | M | 0.160 | 0.840 | M | 0.071 | **0.929** | M | 0.019 | **0.981** | M | 0.089 | **0.911** | M | 0.322 | 0.678 | M | 0.325 | 0.675 | F | 0.594 | 0.406 | M | 0.274 | 0.726 |
| M | 0.368 | 0.632 | M | 0.210 | 0.790 | M | 0.022 | **0.978** | M | 0.180 | 0.820 | M | 0.167 | 0.833 | M | 0.152 | 0.848 | M | 0.211 | 0.789 | M | 0.386 | 0.614 | M | 0.429 | 0.571 | F | 0.692 | 0.308 | M | 0.342 | 0.658 |
| M | 0.368 | 0.632 | M | 0.387 | 0.613 | M | 0.022 | **0.978** | M | 0.174 | 0.826 | M | 0.073 | **0.927** | M | 0.166 | 0.834 | M | 0.304 | 0.696 | M | 0.388 | 0.612 | M | 0.456 | 0.544 | F | 0.666 | 0.334 | M | 0.348 | 0.652 |
| M | 0.263 | 0.737 | M | 0.038 | **0.962** | M | 0.022 | **0.978** | M | 0.204 | 0.796 | M | 0.134 | 0.866 | M | 0.024 | 0.976 | M | 0.064 | **0.936** | M | 0.325 | 0.675 | M | 0.261 | 0.739 | F | 0.517 | 0.483 | M | 0.275 | 0.725 |
| F | 0.684 | 0.316 | F | **0.902** | 0.098 | F | 0.740 | 0.260 | F | 0.686 | 0.314 | F | 0.681 | 0.319 | F | **0.967** | 0.033 | F | 0.898 | 0.102 | F | 0.631 | 0.369 | F | 0.635 | 0.365 | F | 0.817 | 0.183 | F | 0.635 | 0.365 |
| M | 0.053 | **0.947** | M | 0.004 | **0.996** | M | 0.022 | **0.978** | M | 0.176 | 0.824 | M | 0.095 | **0.905** | M | 0 | **1** | M | 0.006 | **0.994** | M | 0.178 | 0.822 | M | 0.147 | 0.853 | M | 0.307 | 0.693 | M | 0.145 | 0.855 |
| M | 0.474 | 0.526 | M | 0.471 | 0.529 | M | 0.022 | **0.978** | M | 0.138 | 0.862 | M | 0.144 | 0.856 | F | 0.594 | 0.406 | M | 0.421 | 0.579 | M | 0.464 | 0.536 | F | 0.503 | 0.497 | F | 0.749 | 0.251 | M | 0.430 | 0.570 |
| M | 0 | **1** | M | 0 | **1** | M | 0.022 | **0.978** | M | 0.114 | 0.886 | M | 0.014 | **0.986** | M | 0 | **1** | M | 0.001 | **0.999** | M | 0.128 | 0.872 | M | 0.072 | **0.928** | M | 0.359 | 0.641 | M | 0.109 | 0.891 |
| M | 0.053 | **0.947** | M | 0 | **1** | M | 0.022 | **0.978** | M | 0.142 | 0.858 | M | 0.024 | **0.976** | M | 0 | **1** | M | 0.001 | **0.999** | M | 0.124 | 0.876 | M | 0.074 | **0.926** | M | 0.221 | 0.779 | M | 0.106 | 0.894 |
| M | 0.105 | 0.895 | M | 0.004 | **0.996** | M | 0.022 | **0.978** | M | 0.184 | 0.816 | M | 0.058 | **0.942** | M | 0 | **1** | M | 0.008 | **0.992** | M | 0.211 | 0.789 | M | 0.165 | 0.835 | M | 0.494 | 0.506 | M | 0.170 | 0.830 |
| M | 0.263 | 0.737 | M | 0.101 | 0.899 | M | 0.022 | **0.978** | M | 0.162 | 0.838 | M | 0.017 | **0.983** | M | 0.038 | **0.962** | M | 0.090 | **0.910** | M | 0.339 | 0.661 | M | 0.269 | 0.731 | F | 0.511 | 0.489 | M | 0.290 | 0.710 |
| M | 0.474 | 0.526 | M | 0.492 | 0.508 | M | 0.022 | **0.978** | M | 0.164 | 0.836 | M | 0.102 | 0.898 | M | 0.231 | 0.769 | M | 0.345 | 0.655 | M | 0.414 | 0.586 | F | 0.501 | 0.499 | F | 0.693 | 0.307 | M | 0.377 | 0.623 |

Table SI 17: Summary table of linear regression analysis results on the accuracy of ML models for the modern and archaeological samples.

| **Model** | **Df** | **Sum Sq** | **Mean Sq** | **F (*p*-value)** |
| --- | --- | --- | --- | --- |
| *Shape*  *Regression*  *Residual* | 1  42 | 790.8  5464.0 | 790.78  130.10 | 6.078 (0.01785) |
| *Form*  *Regression*  *Residual* | 1  42 | 346.92  2438.53 | 346.92  58.06 | 5.9751 (0.01879) |
|  | **R** | **R2** | **Adjusted R Square** |  |
| *Shape* | 11.41 | 0.1264 | 0.1056 |  |
| *Form* | 7.62 | 0.1245 | 0.1037 |  |
|  | **coefficients** | **SE** | **t-Stat (*p*-value)** |  |
| *Shape*  *Intercept*  *Accuracy* | 33.705  32.760 | 9.935  13.288 | 3.393 (0.00152)  2.465 (0.01785) |  |
| *Form*  *Intercept*  *Accuracy* | 42.302  24.018 | 8.342  9.826 | 5.071 (8.45e-06)  2.444 (0.018788) |  |

Table SI 18: Quantification of missing landmarks per specimen.

| **Missing LMs** | **Country** | | **Total** |
| --- | --- | --- | --- |
|  | Portugal | Sudan |  |
| 0 | 53 | 4 | 57 |
| 1 | 9 | 5 | 14 |
| 2 | 5 | 1 | 6 |
| 3 | 0 | 5 | 5 |
| 4 | 0 | 2 | 2 |
| 5 | 0 | 1 | 1 |
| **Total** | 67 | 18 | 85 |

Table SI 19: Landmark dataset used in this study.

| # LM | LM name | Landmark definition |
| --- | --- | --- |
| 1 | Gnathion | Midline of the inferior border of the mandible |
| 2 | Infradentale | On the anterior alveolar ridge, between anterior incisors |
| 3 | Linguale | Genial tubercle: in case of a single tubercle, on its tip; in case of two, midpoint between them |
| 4 | Orale, mandible | On the posterior alveolar ridge between the anterior incisors |
| 5 | Pogonion | Most anterior point of mandibular symphysis |
| 6 | C-P3 | On the anterior alveolar ridge between canine and first premolar |
| 7 | P4-M1 | On the anterior alveolar ridge between second premolar and first molar |
| 8 | M1-M2 | On the anterior alveolar ridge between first and second molar teeth |
| 9 | Mental Foramen Anterior | Anterior point of mental foramen |
| 10 | Ramus Root | On the anterior rim of the ramus (placed on the level of the alveolar ridge) |
| 11 | Gonion | A point on the projection of the bisection of the mandibular angle |
| 12 | Condyle, lateral | From a superior view, the lateral point on the condyle |
| 13 | Condyle, midpoint | From a superior view, a point in the center of the condyle |
| 14 | Condyle, medial | From a superior view, the medial point on the condyle |
| 15 | Sigmoid Notch | Mandible is positioned in the mandibular plane in a lateral view, then the lowest point of the mandibular notch is marked |
| 16 | Coronoid process | Tip of the coronoid process |
| 17 | Mandibular Foramen, inferior | Most inferior point of the mandibular foramen |
| 18 | Alveolous, lingual posterior | From a superior view, the most posterior point on the lingual alveolar process |
| 19 | Condyle, anterior | A point on the antero-superior aspect of the mandibular notch (on the condyle) |
| 20 | Condyle, posterior | The center of the condyle from a posterior view |
| 21 | Ramus, posterior | Posteriormost point of the ramus that is line witht the ramus root |

Table SI 20: Description of the ML algorithms used in the present study.

| **Model** | **Description** |
| --- | --- |
| k-nearest neighbour (kNN) | The algorithm is a non-parametric, supervised learning method used for classification and regression by evaluating the proximity of data points. For classification tasks, labels are assigned based on the majority class among the "k" closest neighbours. In regression, it predicts a value by averaging the target values of these neighbours. The number of neighbours, denoted by "k", is a key parameter impacting accuracy, as is the choice of distance metric. Tuning these parameters helps optimise model performance, typically achieved by fitting KNN to the training set and validating results on the test set. |
| Logistic Regression (LGR) | It establishes a relationship between input variables and an output probability through the logistic (sigmoid) function, which maps predictions to values between 0 and 1, allowing for threshold-based categorisation into binary outcomes. This probability score represents the likelihood that an observation belongs to a specific class. Prediction errors can be minimised through tuning of a loss function that assigns a “cost” to inaccurate predictions and the gradient descent, an optimization algorithm, which iteratively updates model weights to reduce this loss. |
| Decision Trees (DTC5.0) | Decision Trees (DTs) create a branching structure where variables and their attributes define splits, leading to terminal nodes that produce classification outcomes based on specific combinations. This "tree structure" is generated through recursive data partitioning. DTs are non-parametric models designed to minimize residuals without the constraints of parametric tests (e.g., normality or homoscedasticity). This approach enhances decision tree performance, achieving efficiencies comparable to more complex machine learning methods. |
| Random Forest (RF) | The model classifies data by creating multiple independent trees from random subsets of variables using the bagging technique. Each tree is built from a unique set of variables selected from the training set, while the testing set (out-of-bag, OOB observations) validates the model. Bagging combines multiple bootstrap samples, averaging predictions for a final outcome. The OOB error is minimized through iterative estimation, providing a built-in cross-validation mechanism. Variable importance is determined by the mean decrease in error (MDE). |
| Gradient Boosting (GB) | The algorithm builds an ensemble of trees, where each tree learns from the errors of the previous one, improving accuracy through iterative error reduction. By adjusting the learning rate, which affects step size and processing time, a balance is struck between computational demand and predictive accuracy. Using gradient descent optimization, parameters are fine-tuned to minimize the loss function, while stochastic gradient descent adds randomness to avoid local minima. |
| Naïve Bayes (NB) | Probabilistic algorithms based on Bayes' Theorem, commonly used for classification tasks that operate under the assumption of feature independence, meaning each feature contributes to the prediction independently of others. Naive Bayes models estimate the probability of an instance belonging to a particular class based on given feature values. This assumption of feature independence simplifies calculations and can be effective for high-dimensional data. To improve prediction accuracy, smoothing techniques (with an alpha parameter, typically set to 1) are applied to handle zero-probability issues in sparse data distributions. |
| Linear Discriminant Analysis (LDA) | The model offers a dimensionality reduction technique to enhance class separability, effectively distinguishing between two or more classes. It projects features from a higher-dimensional space onto a lower-dimensional space, maximising between-class variance while minimising within-class variance. It assumes that data is Gaussian, covariance matrices across classes are equal, and that classes are linearly separable. The number of resulting dimensions can be adjusted as a parameter to control the degree of dimensionality reduction. |
| Partial Least Squares (PLS) | This algorithm models relationships between independent and dependent variables, particularly when many interrelated predictors are present. It identifies latent variables that explain maximum variance in both sets of variables. By iteratively extracting these variables, it maximises covariance, enabling effective classification in multidimensional data. This method reduces dimensionality and improves interpretability, enhancing accuracy by selecting optimal predictor combinations for class separation. |
| Support Vector Machines (SVM) | SVM algorithms are effective for non-linear multidimensional spaces by dividing data with a hyperplane, which separates classes. This separation is determined by a kernel function. SVM models were tuned using a residual threshold and a cost parameter (C-classification) within the loss function. The cost value, which controls the hyperplane's width, penalizes models with large residuals. Lower cost values create wider margins but may increase misclassification rates, while higher values produce narrower margins for better accuracy. Non-linear kernels can be further optimized with the sigma parameter to adjust convolution effects. |
| Neural Networks (NNET) | The algorithms generate structures based on nodes, mimicking neural networks in the brain, and are organised in hierarchical layers that rely on regression methods. A backpropagation system enables learning and self-correction by adjusting weights, ultimately yielding a final output in a designated node. To reduce the sum of square residuals (SQR) and prevent overfitting, several parameters were optimized, particularly the lambda (λ) value, which controls weight decay across layers, and the number of hidden layers used in the model. |

Table SI 21: Sex estimation of the Jebel Sahaba individuals according to Crevecoeur, et al. ^76^ (the coding in the “Sex” column express reliability in the estimations, which are explained in the original article and were dichotomized in this study). The corresponding machine learning predictions and probabilities are provided in the Table SI 9 to Table SI 16.

| Individual | Sex (Crevecouer et al. 2021) |
| --- | --- |
| JS 10 | pM? |
| JS 15 | pF? |
| JS 16 | pF? |
| JS 17 | pM? |
| JS 19 | M |
| JS 20 | pM? |
| JS 21 | pF |
| JS 22 | M |
| JS 23 | pF? |
| JS 25 | pF? |
| JS 28 | pF |
| JS 29 | M |
| JS 34 | pF? |
| JS 38 | M |
| JS 39 | pM |
| JS 42 | pM |
| JS 102 | pF |
| JS 106 | M |
